# Endo-exo framework for a unifying classification of episodic landslide movements: Implications for forecasting catastrophic failures

**DOI:** 10.1126/sciadv.ady9141

**Published:** 2025-09-24

**Authors:** Qinghua Lei, Didier Sornette

**Affiliations:** ^1^ Department of Earth Sciences, Uppsala University, Uppsala, Sweden.; ^2^ Institute of Risk Analysis, Prediction and Management, Academy for Advanced Interdisciplinary Studies, Southern University of Science and Technology, Shenzhen, China.

## Abstract

Landslides exhibit intermittent downslope movements over days to years before a possible collapse, commonly boosted by external events like precipitation. The origins of these episodic movements and their relation to final instability remain unclear. We propose a novel “endo-exo” theory capturing the interplay between exogenous stressors such as rainfall and endogenous damage-healing processes. We predict four types of episodic dynamics, defined by the disturbance source (endogenous or exogenous) and criticality level (subcritical or critical), each with distinct power law exponent but all governed by a single parameter ϑ . These predictions are tested on the Preonzo landslide, whose sporadic activities align with the classification with ϑ≈0.45±0.1 . We find that its final collapse is preceded by an increased frequency of medium-to-large velocities over 1 to 2 months, signaling the transition into a catastrophic regime. Our research suggests that landslides may not permanently operate at a critical state, with major implications for forecasting catastrophic failures.

## Teaser

Landslides may not remain at criticality: Episodic dynamics shaped by endo-exo interplay reveal key paths to catastrophic failure.

## INTRODUCTION

Landslides, a widespread form of mass wasting, occur in various Earth surface environments and pose great threats to life and property worldwide (*1*, *2*). Because of rapid population growth and urbanization, human habitats are increasingly exposed to landslide hazards, with the situation becoming even more severe under climate change, where extreme rainfall, permafrost thaw, and glacier retreat have promoted fatal landslides (*3*). Extensive field observations show that landslides commonly exhibit episodic movements characterized by intermittent acceleration-deceleration sequences that are boosted by external events like precipitation and earthquakes (*4*–*12*). Some landslides have episodically moved over hundreds or thousands of years without collapse, while others could evolve into a major collapse after episodically deforming over days to years (*13*). The reasons behind these episodic movements (marked by intermittent bursts of displacement activities followed by sustained periods of relaxation dynamics) and how they relate to a possible final catastrophic failure remain poorly understood, inhibiting our capability to predict landslide behavior and mitigate the associated risks.

**Fig. 1.**
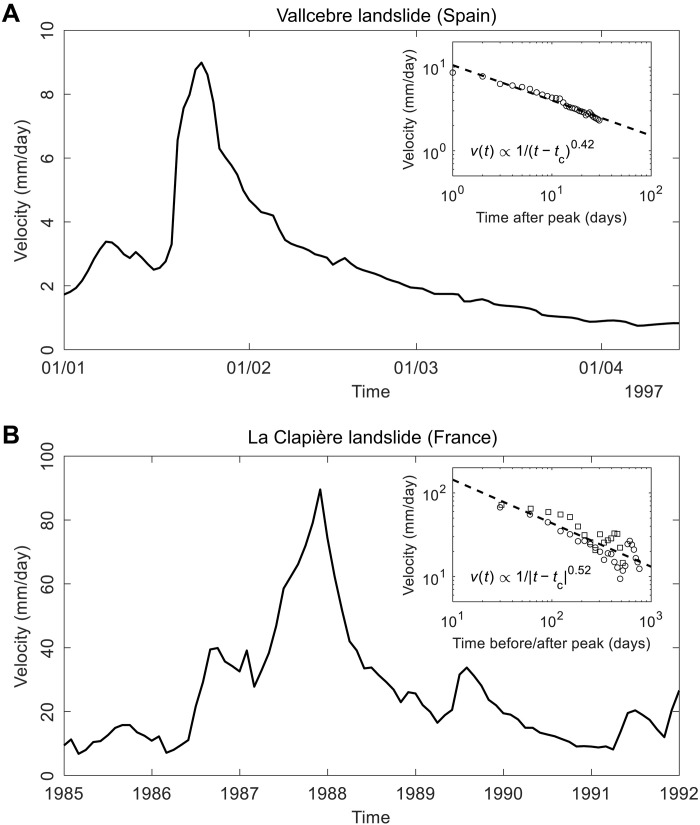
Typical episodic landslide movement behavior around a major velocity peak. (**A**) Velocity time series of the Vallcebre landslide (Spain) around an exogenous velocity peak (associated with rainfall-induced groundwater table fluctuation); inset shows the power law velocity dynamics v(t) after the peak at time $t_{c}$ . (**B**) Velocity time series of the La Clapière (France) around an endogenous velocity peak; inset shows the power law velocity dynamics v(t) before (squares) and after (circles) the peak at time $t_{c}$.

We identify the following fundamental questions: (i) Are episodic landslide movements of an exogenous or endogenous origin? (ii) What are their underlying mechanisms? (iii) How do they relate to catastrophic failures? By exogenous (exo), we refer to external forcing, such as rainfall, snowmelt, seismicity, and anthropogenic activities; by endogenous (endo), we refer to internal processes such as damage, healing, internal faulting, and evolution of frictional sliding properties. Figure 1 presents two typical examples of episodic landslide movements, illustrating the differences in precursory and recovery dynamics around intermittent peaks in velocity. The Vallcebre landslide (Spain) (*14*) shows an exogenous velocity peak (associated with rainfall-induced groundwater table fluctuation), characterized by a sudden jump followed by a gradual recovery that exhibits a power law trend (Fig. 1A). In contrast, the La Clapière landslide (France) (*15*) shows an endogenous peak in velocity, characterized by an almost symmetric gradual increase and decrease in velocity, with both trends following almost the same power law (Fig. 1B). Similar episodic movement patterns have been observed among different types of slope movement, including rockfalls, rockslides, soilslides, and earthflows (see text S1, figs. S1 and S2, and table S1).

**Fig. 2.**
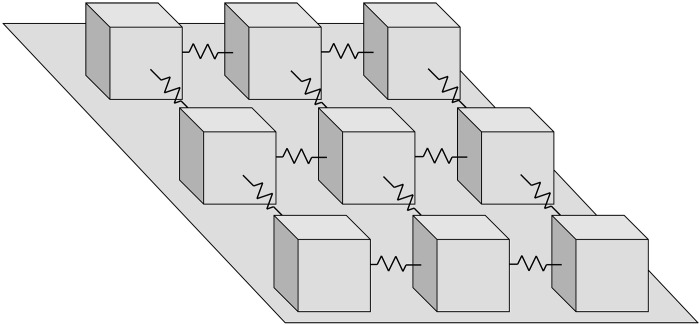
Conceptual picture of a landslide. The landslide as a complex system consists of numerous geomaterial masses (represented by sliders) lying on an inclined substrate and interacting via cohesive or frictional contacts (represented by springs).

We develop a novel “endo-exo” theoretical framework to quantitatively diagnose exogenous and endogenous velocity peaks in episodic landslide movements. In this framework, a landslide is conceptualized as a complex system consisting of numerous geomaterial masses lying on an inclined substrate and interacting via cohesive or frictional contacts (Fig. 2), analogous to the classical spring-slider model applied to earthquakes (*16*) and landslides (*17*). The masses can move and interact under the combined effects of external triggers (such as rainfall and seismicity) and internal processes (such as damage and healing). The emergence of macroscopic landslide motion (observable at geodetic scales) is a natural consequence of the collective behavior of these masses interacting across spatiotemporal scales. On the basis of the principles of statistical physics, we derive analytical solutions that characterize the emergent macroscopic behavior of the landslide.

We apply this framework to investigate the roles of exogenous triggers and endogenous processes in episodic landslide movements before the approach to catastrophic failure. We conduct a case study using the long-term monitoring dataset of a rainfall-induced landslide at Preonzo, in Switzerland. We further explore the mechanisms underlying the transition from episodic movement to catastrophic collapse. Our findings open up a new avenue for understanding landslide dynamics and forecasting catastrophic failures.

## RESULTS

### An epidemic cascade model of mass interactions

The displacement of a landslide results from a combination of external and internal influences, under which a mass may move and disturb its neighbors by redistributing mechanical stress, pore pressure, and other physicochemical conditions. Thus, the initial movement of a mass (the so-called “mother” mass) may trigger movements of its immediate neighbors (the first-generation “daughter” masses) which, in turn, promote movements of their own neighbors, leading to a cascade of movements spreading across the system. Such an epidemic cascading behavior is described by (*18*, *19*)

$$\begin{array}{c} v\left(t\right)=V\left(t\right)+n\int_{-\infty }^{t}\psi \left(t-\tau \right)v\left(\tau \right)d\tau =\\ V\left(t\right)+n\int_{-\infty }^{t}V\left(\tau \right)\Psi \left(t-\tau \right)d\tau \end{array}$$ (1)

where v(t) is the average displacement rate (i.e., velocity) and V(t) represents the exogenous activation (excluding any cascading effect). The parameter n≥0 is the so-called branching ratio, defined as the average number of triggered first-generation daughter masses per mother mass. For n≤1 , it is also the fraction of triggered mass movements over all mass movements; for instance, n≈0.61 (a scenario analyzed later) implies that ~61% of mass movements are endogenously triggered and ~39% are exogenously caused. ψ(t−τ) is the probability distribution function of the waiting time between the movement of a mother mass at time τ and the movement of a first-generation daughter mass at a later time t , while Ψ(t−τ) is the probability of the waiting time between the movement of a mass at time τ and the triggered movement of another mass at a later time t through any possible generation lineage. In other words, ψ(t−τ) governs the triggering within each individual mother-daughter pair, while Ψ(t−τ) considers the cascades through the full genealogy of mother-daughter descendants.

The first equality in Eq. 1 expresses how the present velocity v(t) is influenced by all previous motions via ψ(t−τ) , which governs each individual interaction, with the resulting equation having a self-consistent integral structure. The second equality provides the formal solution of the integral equation, showing that v(t) results from the past exogenous activation V(τ) until the present by Ψ(t−τ) , which incorporates the cumulative effect of all the cascading mass interactions.

We assume that ψ(t−τ) obeys a power law characterizing a long-memory process, (*18*)

$$\psi \left(t-\tau \right)\propto 1/{\left(t-\tau \right)}^{1+\vartheta },\text{with}\;0<\vartheta <1\;\text{and for}\;t-\tau >c$$ (2)

where ϑ controls the persistence of memory (the larger the value of ϑ , the shorter the memory); c is a small characteristic time scale defining the onset of the power law decay and reflecting the rupture time (*20*). This c value may result from brittle creep (*21*), viscous deformation (*22*), pore pressure diffusion (*23*), and/or frictional slip (*24*). Here, the condition ϑ>0 ensures that ψ(t−τ) is normalizable, while the condition ϑ<1 is suggested by empirical observations from the literature (*25*–*29*). The power law form of ψ(t−τ) is supported by many empirical observations such as Andrade’s law of material creep (*30*) and Omori’s law of aftershock activity (*29*), and can be derived theoretically from the constitutive laws of subcritical crack growth (*31*), rate-state friction (*24*), and material rheology (*26*).

The branching ratio n quantifies the extent to which past mass movements endogenously trigger future ones. When n=1 , the system is said to be at criticality (*32*, *33*): Each active mass movement, on average, leads to one subsequent event. However, this average conceals large fluctuations: individual realizations can vary markedly, with cascade sizes following a heavy-tailed power law distribution (*33*). Rather than indicating a steady conservation of activity, the critical point reflects a state of heightened susceptibility, where small perturbations or noise can give rise to disproportionately large avalanches due to the system’s intrinsic instability. If n<1 , the system is in the subcritical regime (*32*, *33*), where there is less than one mass triggered per moved mass on average, such that the number of triggered masses eventually decays to zero; in this regime, the system is dissipative, with the energy released by moving masses smaller than the energy lost for triggering them. If n>1 , the system is in the supercritical regime (*32*, *33*), where the number of triggered masses on average grows exponentially with time (*25*) or even faster (*34*); in this regime, the released energy from a mobilized mass is in general greater than the dissipated energy for mobilizing it.

Here, we focus on the subcritical and critical regimes with n≲1 to ensure stationarity; the transition into the supercritical regime n>1 and catastrophic failure (*25*, *34*) will be explored in Discussion. Note that our subcritical, critical, and supercritical regimes are defined on the basis of the many-body effects of numerous masses interacting and cascading across spatiotemporal scales within a landslide. They are not the same as the regimes defined in the context of fracture mechanics-based slope stability analysis (*35*), where a landslide is treated as a single global mass, with subcritical and critical states distinguished by the relative magnitudes of imposed stress and available strength.

### Classification of episodic landslide dynamics

**Fig. 3.**
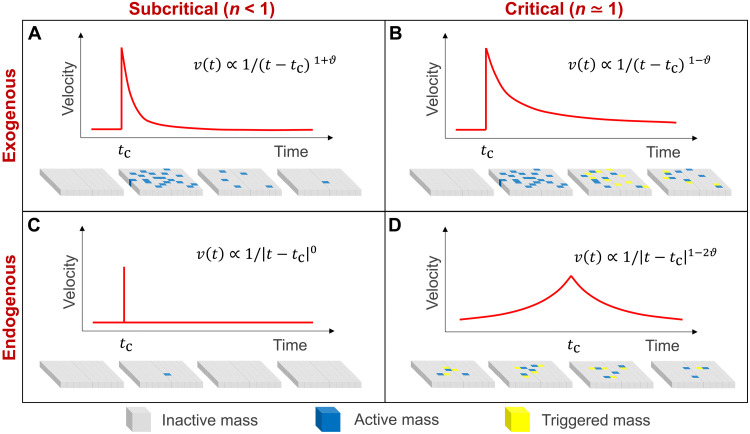
Classification of episodic landslide movements based on the origin of disturbance (endogenous versus exogenous) and the level of criticality (subcritical or critical). There are four distinct types of power law velocity dynamics v(t) around a peak at time $t_{c}$ and they are all related to a single parameter : (**A**) type I, exogenous-subcritical; (**B**) type II, exogenous-critical; (**C**) type III, endogenous-subcritical; (**D**) type IV, endogenous-critical ϑ . The series of snapshots below each velocity trajectory illustrates the evolution of a mass system (with inactive masses, active masses, and triggered masses color-coded) underpinning the velocity time history.

Analytical solutions of Eq. 1 (see Eq. 13 in Materials and Methods) indicate that landslide velocities around a peak at time $t_{c}$ can be described by a generalized finite-time singularity power law as

$$v\left(t\right)\propto 1/\;{|\;t-t_{c}\;|}^{p}$$ (3)

where the exponent p depends on the parameter ϑ and the regime delineated by a characteristic time $t^{*}$ (defined by Eq. 9 in Materials and Methods). This allows us to classify episodic movements into four fundamental types (Fig. 3) based on the source of disturbance (endogenous or exogenous) and the level of criticality (subcritical or critical):

1) Type I: Exogenous subcritical, with p=1+ϑ for $t-t_{c}>t^{*}$ (see Eq. 8 in Materials and Methods). Here, the cascading propensity is limited ( n<1 ), meaning that the exogenously induced velocity burst at time $t_{c}$ does not cascade beyond the first few generations of triggered masses (Fig. 3A).

2) Type II: Exogenous critical, with p=1−ϑ for $c<t-t_{c}<t^{*}$ (see Eq. 8 in Materials and Methods). Here, the system is in a critical state ( n≃1 ), such that the exogenously induced velocity peak at time $t_{c}$ cascades throughout the system, triggering the motion of neighboring masses that further trigger their own neighbors and so on (Fig. 3B).

3) Type III: Endogenous subcritical, with p=0 for $|\;t-t_{c}\;|>t^{*}$ (see Eq. 13 in Materials and Methods). The displacement activity does not result from an exogenous event but instead from endogenous interactions in the presence of small external noisy perturbations. Limited cascading occurs ( n<1 ) such that the (small) peak has no apparent precursory or recovery signatures (Fig. 3C).

4) Type IV: Endogenous critical, with p=1−2ϑ for $c<|\;t-t_{c}\;|<t^{*}$ (see Eq. 12 in Materials and Methods). The displacement activity originates from endogenous growth and interaction within the system in a critical state ( n≃1 ), where the triggering cascades produce an approximately symmetrical power law acceleration-deceleration behavior around the peak (Fig. 3D).

This classification arises from the interplay of the long-memory process (as embodied in Eq. 2) and the epidemic cascade throughout the system (as captured by Eq. 1). The four peak types, associated with distinct power law exponents 1+ϑ , 1−ϑ , 0 , and 1−2ϑ , are all governed by a single parameter ϑ , highlighting the unifying nature of our classification. Notably, while the type III peak has a constant exponent of 0 , it remains formally integrable into this ϑ-governed family of exponents as a constant function. It can be seen that the relaxation following an endogenous-critical peak (with a smaller exponent p=1−2ϑ ) is slower than that following an exogenous-critical peak (with a larger exponent p=1−ϑ ). This longer-lived influence of an endogenous-critical peak results from the precursory process that impregnates the system much more than its exogenous counterpart (*19*). Note that exogenous peaks lack apparent precursors, because they are caused by external events that act as sudden shocks to the system. The distinct properties of different peak types enable differentiation between endogenous and exogenous origins of episodic landslide movements, based on the system’s response around peaks rather than (often elusive) correlations with external events. Accordingly, measurements of external events such as rainfall or seismicity are not required to infer the origin of a peak, although they can serve as useful corroborating evidence. The differentiation here concerns whether the observed peak is endogenous or exogenous, based on its pre- and postpeak dynamics, rather than on the system’s internal mechanisms, which remain fundamentally the same in both cases.

### Application to the Preonzo landslide, Switzerland

**Fig. 4.**
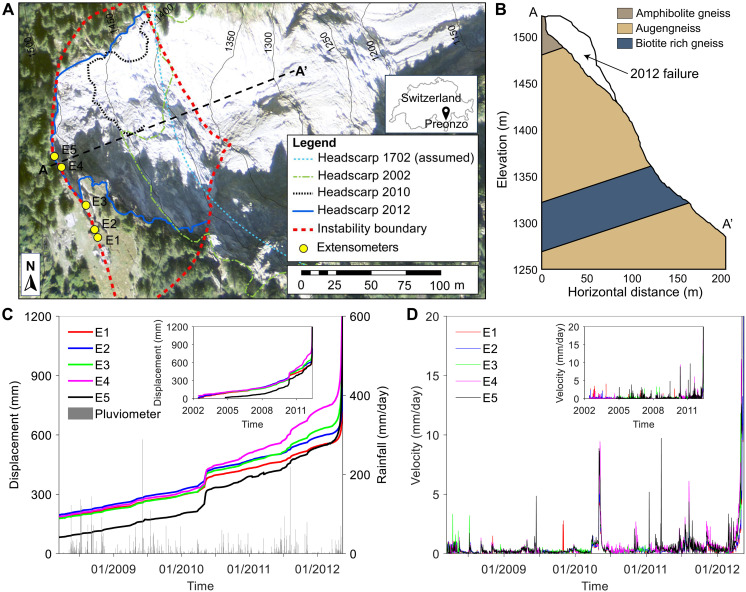
Preonzo landslide, Switzerland. (**A**) Overview of the landslide site with the locations of five extensometers E1 to E5, the boundary of this instability complex, the headscarps of historical failure events, and the cross-section line A-A’ indicated. (**B**) Cross-section A-A’ of the slope with the lithological units and the topographies (before and after the 2012 failure event) depicted. (**C**) Monitoring data of slope displacements by the five extensometers and recorded data of daily rainfall amount by a pluviometer installed at the slope. (**D**) Slope daily velocity time series derived from the displacement data.

We test our theory based on the long-term monitoring dataset of a rainfall-induced landslide above the village of Preonzo, Switzerland (*36*) (Fig. 4A), which exhibited notable episodic movements over many years before a catastrophic failure in 2012. This instability complex is situated on a steep slope (dipping about 60° toward the Riviera valley), which is mainly composed of amphibolite gneiss, augengneiss, and biotite-rich gneiss (Fig. 4B). Extensive fractures divide the slope into numerous rock blocks (*37*), consistent with our conceptual model (Fig. 2). The landslide has experienced multiple failures since the 18th century (*37*): The collapse in February 1702 destroyed the ancient village of Preonzo; in May 2002, ~120,000 m^3^ of rock from the southern sector was detached; in May 2010, ~20,000 m^3^ of rock from the northern sector was released; in May 2012, ~210,000 m^3^ of rock catastrophically failed (Fig. 4A shows the headscarps of these historical events). The 2012 failure occurred in a mixed kinematic mode, involving oblique and flexural toppling in the headscarp region, and planar and wedge sliding along the basal rupture plane formed by preexisting fractures and new cracks (*37*).

The landslide posed a great threat to the industrial and transport infrastructures located directly at the toe of the slope (*36*). Five high-precision extensometers E1 to E5 (Fig. 4A) were installed to measure the opening of tension cracks in the headscarp area. From 2008, a pluviometer was installed to monitor the local precipitation. Figure 4C shows the time series of slope displacement measured by the five extensometers and of daily rainfall amount recorded by the pluviometer between 2008 and 2012 (see fig. S3 for the time series of rainfall intensity and cumulative rainfall). Daily velocities computed from the displacement data (Fig. 4D) show a step-like behavior over time (Fig. 4C) with episodic short-term movements superimposed on a long-term deformation trend that shifts from deceleration (2002 to 2006) to acceleration (2006 to 2012) (figs. S4 and S5).

**Fig. 5.**
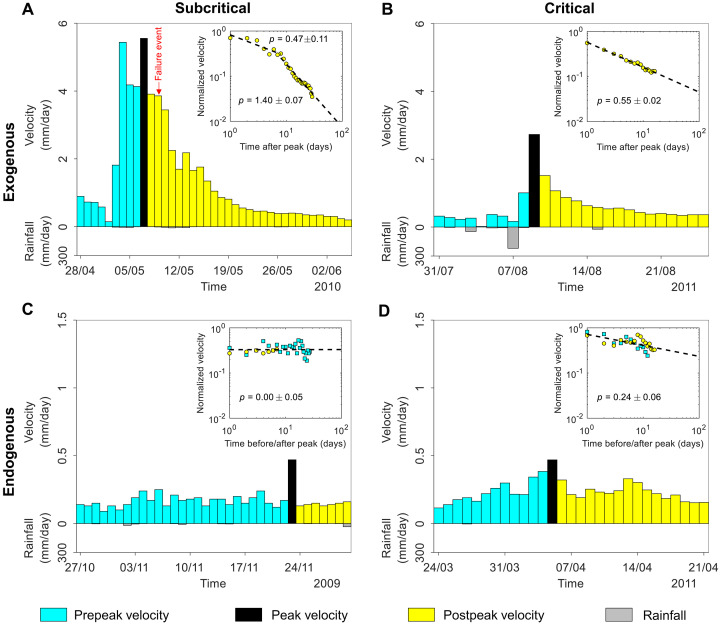
Four categories of episodic landslide dynamics found in the velocity time series of the Preonzo landslide. (**A**) Type I, exogenous subcritical; (**B**) type II, exogenous critical; (**C**) type III, endogenous subcritical; and (**D**) type IV, endogenous critical. Here, (A), (B), and (D) show velocity data from extensometer E1, and (C) shows velocity data from extensometer E5. The red arrow in (A) marks the timing of the local failure of a downslope northern sector of the slope on 9 May 2010. Insets show the postpeak relaxation of normalized velocity where dashed lines indicate the power law fitting [in the insets of (C) and (D), prepeak velocity data of those endogenous peaks are also included in the fitting].

The velocity time series show all four types of episodic dynamics (Fig. 5 and figs. S6 to S10). In each case, we estimated the exponent p by fitting normalized velocities to a power law and derived its standard deviation from the confidence interval of the fit (see Materials and Methods).

For the type I exogenous-subcritical peak on 7 May 2010 (Fig. 5A; extensometer E1), the velocity relaxation beyond ~8 days after the peak is characterized by an exponent of p=1.40±0.07 (exogenous subcritical), whereas its early-time relaxation within ~8 days is associated with a much smaller exponent of p=0.47±0.11 (exogenous critical), as expected from the prediction by Eq. 3. All five extensometers exhibit a similar two-branch power law relaxation behavior with p=0.46±0.10 for the early-time response and p=1.54±0.06 for the late-time response (figs. S6A and S7). Around this peak with little precipitation (Fig. 5A; see the rainfall record), the slope experienced a localized failure in its northern sector downhill from the tension cracks where the extensometers are installed (Fig. 4A).

The type II exogenous-critical peak on 9 August 2011 occurred 2 days after a heavy rainstorm (exceeding 200 mm/day) on 7 August 2011 (Fig. 5B; extensometer E1 and rainfall record) and was followed by a power law reduction in velocity with p=0.55±0.02 (Fig. 5B, inset). All five extensometers have captured this peak followed by a power law relaxation with p=0.63±0.03 (figs. S6B and S8).

A type III endogenous-subcritical peak occurred on 23 November 2009 (Fig. 5C; extensometer E5). It was not preceded by rainfall. This peak is surrounded by an essentially noisy stationary velocity trajectory with p≈0 (Fig. 5C, inset), whereas most extensometers do not capture this peak and only show random fluctuations (figs. S6C and S9).

A type IV endogenous-critical peak occurred on 05 April 2011 (Fig. 5D; extensometer E1). It was preceded by a progressively accelerating power law growth of velocity and followed by an approximately symmetrical power law relaxation, with a common exponent of p=0.24±0.06 . Most of the five extensomers captured the approximately symmetrical precursory-recovery dynamics with a small power law exponent of p=0.21±0.04 (fig. S6D), although the timings of the peaks were not fully synchronized (fig. S10). One can notice that the time-dependent signatures of endogenous peaks are less apparent compared to exogenous ones, as expected from the small exponent values.

**Fig. 6.**
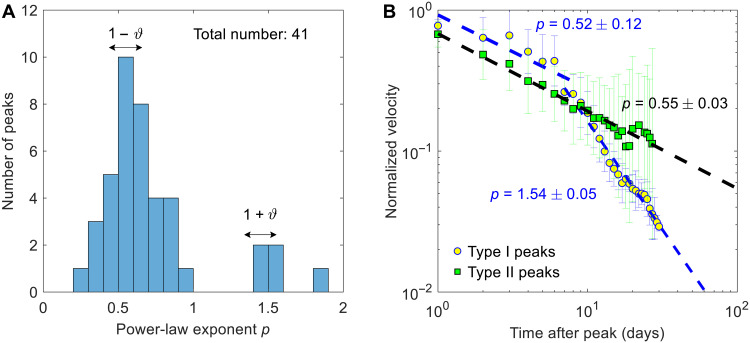
Postpeak relaxation properties associated with detected peaks in the velocity time series. (**A**) Histogram of power law exponents p for postpeak velocity relaxation; the double arrows indicate the value ranges of p=1−ϑ (type II peaks) and p=1+ϑ (type I peaks), with ϑ≈0.45±0.1 . (**B**) Ensemble averaged velocity relaxation behavior for types I and II peaks; error bars indicate the standard deviation associated with the ensemble average. The fitted power laws are consistent with the existence of a single parameter ϑ≈0.45±0.10 . This is particularly revealed by the equality (within statistical fluctuations) of the exponent of type I peaks for $\;t-t_{c}<t^{*}$ ( p=0.52±0.12 ) and of the exponent of type II peaks ( p=0.55±0.03).

A remarkable feature of these examples is that their values of p are all consistent with the presence of a single parameter ϑ≈0.45±0.10 . To investigate whether similar values of ϑ characterize the general behavior of the Preonzo landslide, we developed an automated detection algorithm to identify velocity peaks with well-defined precursory and recovery characteristics from the 10-year long-term monitoring dataset (Materials and Methods). A histogram of power law exponent p shows a clear cluster around 0.59 (for type II peaks) and a second possible cluster at 1.52 (for type I peaks), which are consistent with ϑ≈0.45±0.10 . This was further confirmed by calculating the ensemble average of the relaxation behavior for these two exogenous peaks (Fig. 6B). Here, endogenous peaks (types III and IV with p≈0 and 0.10±0.20 , respectively) are absent, because they have small magnitudes and postpeak responses that fluctuate considerably (Fig. 5, C and D, and fig. S6, C and D) and therefore do not pass the peak detection criteria (Materials and Methods).

## DISCUSSION

Our analysis of the Preonzo slope’s episodic movements has revealed four distinct regimes of precursory and recovery signatures, sharing a common value for ϑ of 0.45±0.10 (Figs. 5 and 6). These regimes distinguish both the source of disturbance (exogenous or endogenous) and the level of criticality (subcritical or critical). Many large velocity peaks of the Preonzo landslide are exogenous critical (Fig. 6), suggesting that the landslide’s behavior in reaction to strong external events (e.g., heavy rainfalls) is often driven by cascades across multiple generations of mass movement triggers. In addition, we have documented a unique exogenous-subcritical peak (Fig. 5A and fig. S6A), which is related to the local failure of a downslope sector (*36*, *37*) on 9 May 2010. Before showing a rapid exogenous-subcritical relaxation, the landslide has actually experienced ~8 days of relatively slower exogenous-critical relaxation. Substituting this characteristic time $t^{*}\approx 8$ days together with ϑ≈0.45 into Eq. 9 (Materials and Methods) and the estimate c≈1 day (text S3), we obtain n≈0.61 . This comparatively low branching ratio aligns with the fact that this local failure did not cascade into a system-sized collapse. However, this local failure brought the system closer to criticality, promoting exogenous-critical peaks (fig. S13) and facilitating the final collapse. In our dataset, we also observe the presence of endogenous-critical peaks (Fig. 5D). However, they are usually associated with small-magnitude peaks and weak time dependence, making them sometimes difficult to be discriminated from the endogenous-subcritical peaks driven by random fluctuations.

The consistent value of ϑ≈0.45±0.10 observed for the Preonzo landslide suggests that its apparently diverse episodic dynamics stem from a common underlying mechanism: cascading mass interactions coupled with long-memory triggering, as captured by our endo-exo formulation (Eq. 2). Recall that ϑ controls the persistence of memory in the triggering process between mother mass movements and their directly triggered daughter mass movements: A smaller ϑ implies that past events exert a longer-lasting influence on future activity in first-generation triggering, whereas a larger ϑ corresponds to a shorter memory, with first-generation triggering dominated by more recent events. A similar value of ϑ≈0.5 has been reported for shallow earthquakes (*38*). A random walk model characterizing stress fluctuations (*39*) offers a plausible explanation for this ϑ value. Consider that, at the moment when a mother mass stops moving, the stresses in its immediate neighbors (the first-generation daughter masses) are marginally lower than the critical threshold (i.e., strength). If the subsequent stress at each first-generation daughter mass fluctuates as a result of random increments (stemming from various sources, such as small-scale damage and healing, poroelastic reorganization, and small earthquakes or rainfall events), the stress dynamics can be described by a Brownian random walk (fig. S11A). The waiting time for a daughter mass to start moving is then determined by the first time its stress exceeds the strength. This “first-passage” time is distributed according to a probability density function asymptotically converging to a power law $\psi \left(t\right)\propto t^{-3/2}$ (*40*), which yields ϑ=0.5 (fig. S11B). This first-passage model also provides a natural interpretation to the origin of parameter c in Eq. 2: It reflects the typical time required for random walkers to cross the gap between the initial stress level and the critical threshold. The smaller the gap, the lower the value of c.

We construct the time history of the branching ratio n for the Preonzo landslide (text S3 and fig. S12B). Compared to the long-term deformation trends (figs. S4 and S5), we interpret that the Preonzo landslide transitioned from healing-dominated condition before 2006 to damage-dominated condition after 2010, driven by gravity-induced creep and precipitation-induced degradation. The evolution of n indicates that this landslide remained in the subcritical regime ( n<1 ) for many years and only approached the critical regime ( n≃1 ) during the final 1 to 2 years. In the last few weeks before the 2012 collapse, our estimates yield n>1 , signaling a transition into the supercritical regime. This behavior, consistent with the recent finding on earthquake faults (*41*), contradicts the conventional concept of self-organized criticality (*42*), which assumes that a landslide always evolves at a critical state, with all events generated from the same underlying process, thereby rendering the prediction of large events impossible. Our results imply that characteristic signatures may emerge during transitions between different regimes, providing warnings of imminent catastrophic events.

**Fig. 7.**
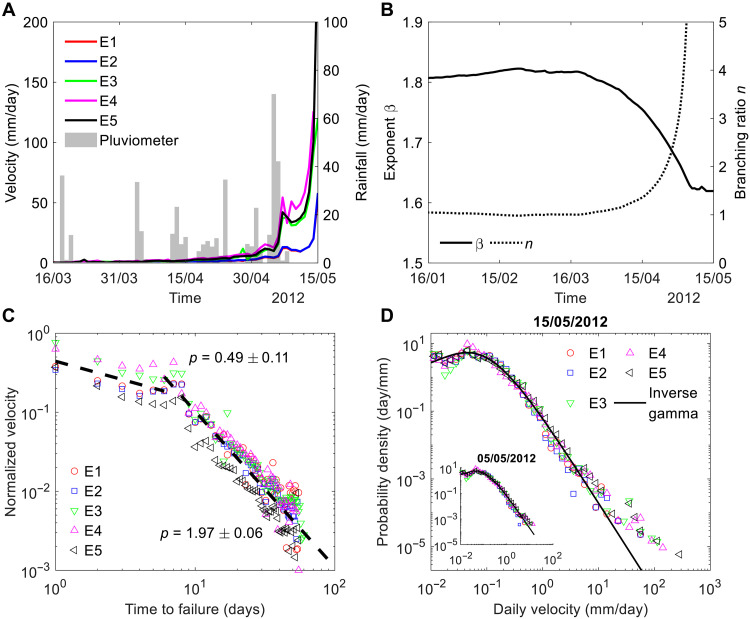
Evolution of the Preonzo landslide before its major collapse on 15 May 2012. (**A**) Time series of the slope velocity measured by the five extensometers E1 to E5 as well as rainfall intensity data recorded by the pluviometer. (**B**) Temporal evolution of the β value of the slope velocity probability distribution and the branching ratio n , which characterizes the internal state of the landslide. (**C**) Variation of normalized velocity before the catastrophic failure as a function of time to the failure (time flows from right to left), which is fitted to a two-branch finite-time singularity power law based on Eq. 3 (indicated by the dashed line). (**D**) Probability density function of daily velocities available until 15 May 2012 (inset, 06 May 2012) fitted to the inverse gamma distribution, where some large velocities manifest themselves as outliers deviating from the inverse gamma.

The endo-exo framework applies to the subcritical and critical regimes ( n≲1 ), where endogenous and exogenous origins of a velocity peak can be distinguished (see text S2). Similarly, the estimation of the branching ratio n (see text S3) is formally valid for n≲1 , but it can serve as an indicator of convergence toward criticality and a potential cross-over into the supercritical regime ( n>1 ), where the system response has an entangled endo-exo origin (see text S2) due to the repetitive interactions and positive feedbacks (*43*). This rationalizes the ambiguous cause-effect relationship between the last rainfall event in early May 2012 and the final collapse of the Preonzo landslide about 10 days after (*36*) (Fig. 7A). Our estimates of n indicate that the landslide approached and eventually entered the supercritical regime characterized by a high degree of endogeneity, rendering it inherently fragile and sensitive to external perturbations. The last rainfall then served as the “the straw that broke the camel’s back” through noise amplification driven by endogenous positive feedbacks (*44*). Near and within the supercritical regime, the assumption of linearity and additivity underlying our epidemic cascade model (Eq. 1) breaks down. A natural extension is provided by nonlinear cascade models (*45*, *46*), in which the event intensity depends not only on the sum of past events but also on nonlinear interactions among them.

Despite these nonlinear effects, landslide velocities in this regime still follow the generalized finite-time singularity power law (Eq. 3) (*43*, *44*, *47*–*49*). We fit the velocity time series of the Preonzo landslide during the final 2 months preceding collapse (Fig. 7A) to Eq. 3 and find it necessary to consider two power law branches, one with p≈1.97 for the early stage and the second one with p≈0.49 for the late stage (Fig. 7C). The values of p observed here, which differ from unity as assumed in the traditional inverse velocity method (*50*), align with extensive field evidence reporting similar deviations (*36*, *48*, *49*, *51*). The transition from p>1 to p<1 reflects a strengthening of positive feedback mechanisms as failure approaches (*44*, *47*). The late-stage large velocities (associated with p≈0.49 ) show statistically different properties, appearing as outliers with respect to other smaller velocities, which follow an inverse gamma distribution characterized by a power law tail exponent β (Fig. 7D and text S4). Such a two-branch time-to-failure power law behavior has also been observed in the ground deformational response before the volcanic eruption at Mount St Helens (*50*). Notably, the Preonzo landslide once experienced a temporary deceleration during 7 to 11 May 2012 just before the final collapse (fig. S14). Similar precursory quiescence phenomena have been observed before some great earthquakes (*52*) and may be attributed to dilatancy hardening (*53*), where pore pressure temporarily drops due to the pronounced dilation effect near the critical crack density (*54*). This dilatancy may result from crack coalescence in rock bridges and/or frictional sliding along rough fractures, compatible with the field observation that the basal rupture plane is formed by preexisting fractures and new cracks (*37*).

The β value of the slope velocity distribution progressively drops from 1.82 to 1.62 over 1 to 2 months before the final collapse (Fig. 7B), indicating an increased occurrence of medium-to-large velocities during the system’s subcritical-to-critical regime transition (text S3). The β value decline before catastrophic landslides is similar to the observed b value decline before great earthquakes (*52*), which is possibly due to increased differential stresses on rock bridges (*55*) and fault patches (*56*). It also finds a natural explanation in the context of cascading triggered events described by self-excited conditional point processes (*57*). This observation points to the possibility to forecast catastrophic landslides by monitoring the decline of the β value (or the increase of the n value) that may occur over a long term (say 1 to 2 months or even longer; Fig. 7B and fig. S12), in addition to detecting the emergence of velocity outliers (Fig. 7D) that may appear over a short term (say 1 to 2 weeks) (*47*).

Our novel endo-exo framework points to a deep quantitative relationship between episodic landslide movements, external triggering events, and internal damage and healing processes within the landmass. The results and insights obtained are of substantial value for landslide prediction, from both the conceptual and operational points of view. Our framework, validated using the Preonzo landslide data, can be applied to many other landslides showing similar episodic movements (*4*–*10*). While our framework incorporates assumptions consistent with empirical observations and is tested against real-world data, it is fundamentally grounded in physics. The broad applicability of our endo-exo solution, i.e., Eq. 3, across various landslide sites and types is supported by the analyses shown in Fig. 1 and figs. S1 and S2. Our observation of the decrease of exponent β before catastrophic failures is consistent across various landslide sites and types, including rockfalls, topples, rockslides, and soilslides (see figs. S15 and S16 and table S1). This metric can be easily calculated in real time by fitting slope velocity data to the inverse gamma function using least squares or maximum likelihood methods, and readily integrated into early warning systems. Last, it is worth noting that the established endo-exo framework has far-reaching implications for forecasting various geohazards, including not only landslides, but also earthquakes, rockbursts, volcanic eruptions, and glacier breakoffs, which all exhibit similar episodic deformations and sometimes also show transitions into catastrophic failures.

## MATERIALS AND METHODS

### Mean field solution of the epidemic cascade model of mass interactions

Consider a system that is subjected to a strong external shock with the exogenous activation V(t) taking the form of a delta function

$$V\left(t\right)=V_{c}\delta \left(t-t_{c}\right)$$ (4)

where $V_{c}$ is the amplitude of the impulse occurring at time $t_{c}$ and $\delta \left(t-t_{c}\right)$ is the unit impulse. Here, V(t) represents the effect of a strong external event that causes a sudden velocity response in the system. The conversion from external forces to V(t) could occur through changes of the system’s internal state (e.g., decreasing the resisting strength via effective stress reduction or material property alteration) and/or changes of the system’s boundary condition (e.g., increasing the driving stress via load application or stress redistribution).

By substituting Eq. 4 into the second integral of Eq. 1, we can obtain

$$\begin{array}{c} v\left(t\right)=V_{c}\delta \left(t-t_{c}\right)\\ +n\int_{-\infty }^{t}V_{c}\delta \left(\tau -t_{c}\right)\Psi \left(t-\tau \right)d\tau =nV_{c}\Psi \left(t-t_{c}\right),\text{for}\;t>t_{c} \end{array}$$ (5)

This expresses that the postpeak dynamics is fully controlled by $\Psi \left(t-t_{c}\right)$ , which is the Green function of the first equality of Eq. 1 (*18*), solution of

$$\begin{array}{c} \Psi \left(t-t_{c}\right)=V_{c}\delta \left(t-t_{c}\right)+n\int_{-\infty }^{t}\psi \left(t-\tau +t_{c}\right)\Psi \left(\tau -t_{c}\right)d\tau \end{array}$$ (6)

For the power law form of ψ(t−τ) as defined in Eq. 2 and considering the normalization constraint $\int_{\tau +c}^{+\infty }\psi \left(t-\tau \right)\mathrm{dt}=1$ , we have

$$\psi \left(t-\tau \right)=\vartheta c^{\vartheta }/{\left(t-\tau \right)}^{1+\vartheta },\text{with}\;0<\vartheta <1$$ (7)

Then, the solution of Eq. 6 for $\Psi \left(t-t_{c}\right)$ can be obtained (*25*) by taking the Laplace transform of Eq. 6, which gives the Laplace transform of $\Psi \left(t-t_{c}\right)$ , and then taking its inverse Laplace transform to yield

$$\begin{array}{c} v\left(t\right)\propto \Psi \left(t-t_{c}\right)\propto \left\{ \begin{array}{c} 1/{\left(t-t_{c}\right)}^{\;1-\vartheta },\text{for}\;c<t-t_{c}<t^{*}\\ 1/{\left(t-t_{c}\right)}^{\;1+\vartheta },\text{for}\;t-t_{c}>t^{*}\; \end{array}\right. \end{array}$$ (8)

where $t^{*}$ is a characteristic time (*25*) given by

$$t^{*}=c{\left[\frac{n\Gamma \left(1-\vartheta \right)}{|\;1-n\;|}\right]}^{\;1/\vartheta }$$ (9)

where Γ(·) is the gamma function. As n→1 (critical regime), $t^{*}\to +\;\infty$ , so that the early-time response prevails ( $t-t_{c}<t^{*}$ ); if 0<n<1 (subcritical regime), $t^{*}$ has a finite value and the system may manifest a coexistence of both early-time response ( $t-t_{c}<t^{*}$ , where epidemic cascades thrive), and late-time response ( $t-t_{c}>t^{*}$ , where cascades become exhausted).

In the absence of any strong external event, a peak in landslide velocity may spontaneously emerge due to the interplay of a continuous stochastic flow of small external perturbations (introducing “noises” into the system) and the amplifying impact of the epidemic cascades of endogenous interactions. The pre- and postpeak average velocity trajectory, conditioned on the existence of an endogenous peak $v_{c}$ at time $t_{c}$ , is given by (*18*)

$$v\left[t\;|\;v\left(t_{c}\right)=v_{c}\right]\approx \frac{v_{c}\;}{\text{var}\left(v_{c}\;\right)}\text{cov}\left[v\left(t\right),v_{c}\right]$$ (10)

for both $t<t_{c}$ and $t>t_{c}$ . Since

$$\begin{array}{c} \text{cov}\left[v\left(t\right),v_{c}\;\right]=\int_{-\infty }^{\text{min}\left(t,t_{c}\right)}\Psi \left(t-\tau \right)\Psi \left(t_{c}-\tau \right)d\tau \end{array}$$ (11)

and $\Psi \left(t-t_{c}\right)\propto 1/{\left(t-t_{c}\right)}^{\;1-\vartheta }$ for $c<|\;t-t_{c}\;|<t^{*}$ , the following solution can be finally obtained (*18*)

$$\begin{array}{c} v\left(t\right)\propto \int_{-\infty }^{\text{min}\left(t,t_{c}\right)}\\ \Psi \left(t-\tau \right)\Psi \left(t_{c}-\tau \right)d\tau \propto 1/{|\;t-t_{c}\;|}^{1-2\vartheta },\text{for}\;c<|\;t-t_{c}\;|<t^{*} \end{array}$$ (12)

or equivalently for n→1 (critical regime). If n<1 (subcritical regime), the system response is essentially a noise process largely driven by random fluctuations, described by

$$v\left(t\right)\propto 1/{|\;t-t_{c}\;|}^{0},\text{for}\;|\;t-t_{c}\;|>t^{*}$$ (13)

### Automated velocity peak detection and calculation of normalized velocities

We implement a peak detection algorithm to automatically extract slope velocity peaks together with their surrounding time series from the 10-year long-term monitoring dataset. We qualify a peak in the velocity time series as a local maximum over a 20-day time window, which is at least k=2.5 times larger than the average velocity over a 2-month time window. The time window sizes and threshold value k are chosen to give an effective detection of good-quality peaks (fig. S17), but the results do not substantially change by varying these parameters (figs. S18 to S24). In addition, we request that each peak has at least 10 days of postpeak data before reaching the next peak. In total, our algorithm detects 330 peaks from the entire dataset recorded by five extensometers. We then fit the postpeak velocity data of each detected peak to a power law over a time window ranging from 10 to 30 days, with the “best” window chosen as the one giving the highest coefficient of determination $R^{2}$ . For the results shown in Fig. 6, we only keep the peaks with $R^{2}>0.8$ to extract unambiguous postpeak response functions, leaving 41 peaks. Our results do not qualitatively change by varying the k threshold from 1.5 to 3.5 and the $R^{2}$ threshold from 0.7 to 0.9 as well as the window sizes for peak detection (figs. S22 to S24), suggesting that our method and results are robust. Despite the limited number of type I peaks in our results (Fig. 6), the estimated median of their exponents p remains robust when varying the peak detection parameters (figs. S22 to S24).

We compute normalized slope velocities $\tilde{v}\left(t\right)$ around a peak based on the following equation

$$\tilde{v}\left(t\right)=\left[v\left(t\right)-v_{r}\right]/\left(v_{c}-v_{r}\right)$$ (14)

where $v_{c}$ is the peak velocity at time $t_{c}$ and $v_{r}$ is the residual velocity when the slope has fully recovered from external perturbations. However, the determination of $v_{r}$ is subject to uncertainties, because a real-world slope rarely has the opportunity to fully recover from one external event (e.g., rainfall) before the next occurs. In this work, we estimate $v_{r}$ by first detecting troughs in the velocity time series. We qualify a trough in the velocity time series as a local minimum over a 20-day time window which is at least k=2.5 times smaller than the 2-month average velocity. The time window sizes and the threshold value k are chosen to give an effective and reasonable detection of troughs (fig. S17), but the results do not substantially change by varying these parameters (figs. S18 to S24). We then define $v_{r}$ associated with a given peak as the minimum of the two nearest troughs (with one before the peak and one after the peak). Note that $v_{r}$ tends to vary over time reflecting the nonstationary characteristic of the landslide. We show the probability density function of calculated $v_{r}$ values in fig. S25 (associated with the identified peaks in fig. S17), which have a mean of 0.008 mm/day. We have tested alternative approaches for determining $v_{r}$ such as using the average of the 10 nearest troughs around a peak or the minimum/average of the troughs located between the former and latter peaks; no substantial changes in the results are found.

### Power law calibration of velocity time series around a peak

We fit the time series of normalized velocities $\tilde{v}\left(t\right)$ around a peak to the finite-time singularity power law function

$$\tilde{v}\left(t\right)=A/\;{|\;t-t_{c}\;|}^{p}$$ (15)

where $t_{c}$ is the time of velocity peak, A is a constant, and p is the power law exponent. To estimate A and p , we use the method of least squares to minimize the sum of squared residuals

$$s=\sum_{t_{i}}\varepsilon {\left(t_{i}\right)}^{2}$$ (16)

with each residual calculated as

$$\varepsilon \left(t_{i}\right)=\text{log}\tilde{v}\left(t_{i}\right)-\text{log}A+p\text{log}|\;t_{i}-t_{c}\;|$$ (17)

We then set the partial derivatives ∂s/∂(logA) and ∂s/∂p to be both zero, leading to solve a linear system of two equations with the two unknowns A and p.

## Supplementary Materials

### This PDF file includes:

Supplementary Text S1 to S4 Figs. S1 to S25 Table S1 References
