# Supplementary material for: Endo-exo framework for a unifying classification of episodic landslide movements: Implications for forecasting catastrophic failures
Source: Sci Adv. 2025 Sep 24;11(39):eady9141. doi: 10.1126/sciadv.ady9141 (PMC13142752; doi:10.1126/sciadv.ady9141)
Supplement: Supplementary file 1 — Supplementary Text S1 to S4 Figs. S1 to S25 Table S1 References [file sciadv.ady9141_sm.pdf]

Science Advances
AAAS

Supplementary Materials for

Endo-exo framework for a unifying classification of episodic landslide movements: Implications for forecasting catastrophic failures

Qinghua Lei and Didier Sornette

Corresponding author: Qinghua Lei, qinghua.lei@geo.uu.se

Sci. Adv. **11**, eady9141 (2025)
DOI: 10.1126/sciadv.ady9141

This PDF file includes:

Supplementary Text S1 to S4
Figs. S1 to S25
Table S1
References

Supplementary Text

Text S1: Endogenous and exogenous peaks across different types of landslides

We compile the monitoring data of 25 landslides, covering different landslide types including rockfalls, topples, rockslides, soilslides, and earthflows, recorded by different instruments (e.g., extensometers, reflectors, distometers, inclinometers, satellites, continuous GPS stations, and synthetic aperture radar) (see table S1 for more details about their locations, types, materials, volumes, and monitoring methods).

In fig. S1, we show a number of exogenous peaks observed in various landslides. Specifically, fig. S1A shows a rainfall-induced exogenous-critical peak at the Veslemannen rockslide (Norway) (*58*); fig. S1B shows an exogenous-critical peak induced by a temperature rise at the Twain Harte granitic exfoliation dome (USA) (*59*); fig. S1C shows a rainfall-induced exogenous-critical peak at the La Saxe rockslide (Italy) (*60*); fig. S1D shows a rainfall-induced exogenous-critical peak at the Pomarico soilslide/earthflow (Italy) (*61*); fig. S1E shows an earthquake-induced exogenous-critical peak at the Maca soilslide (Peru) (*10*); fig. S1F shows an earthquake-induced exogenous-subcritical peak at the Tapgaon rockslide (Nepal) (*62*); fig. 1G shows a rainfall-induced exogenous-subcritical peak at the Yaoshan soilslide (PR China) (*63*); fig. S1H shows a rainfall-induced exogeneous-subcritical peak at the Hollin Hill rockslide (*64*).

In fig. S2, we further present a number of endogenous peaks observed in various landslides. Specifically, fig. S2A shows an endogenous-critical peak at the Veslemannen rockslide (Norway) (*58*); fig. S2B shows an endogenous-critical peak at the Twain Harte granitic exfoliation dome (USA) (*59*); fig. S2C shows an endogenous-critical peak at the Moosfluh rockslide (Switzerland) (*65*); fig. S2D shows an endogenous-critical peak at the Shuping soilslide (PR China) (*66*); fig. S2E shows an endogenous-critical peak at the Oak Ridge earthflow (*7*); fig. S2F shows an endogenous-critical peak (associated with a rockfall event) at the Ingelsberg rock slope (Austria) (*67*); fig. S2G and 2F respectively shows an endogenous-critical peak and an endogenous-subcritical peak at the Séchilienne rockslide (France) (*68*).

These examples demonstrate the prevalence of endogenous and exogenous peaks in various types of landslides across diverse geological sites worldwide. While these examples were selected for illustration, further examples can be found in the dataset.

Text S2: Distinguishing endogenous versus exogenous origins

In a system operating within the subcritical or critical regime, it is possible to differentiate between endogenous and exogenous origins of system responses. This distinction has been demonstrated across various contexts in the literature (*19*). This differentiation relies on the fact that a complex system responds differently to exogenous shocks compared to endogenous peaks, as demonstrated in our analytical solutions (Eqs. 8, 12, and 13 in Materials and Methods), as schematically illustrated in Fig. 3, and as further demonstrated in our application to the Preonzo landslide (Fig. 5 and fig. S6). Here, we focus on the subcritical and critical regimes for the endo-exo classification.

It is important to emphasize that endogenous and exogenous factors are always interconnected, provided that the system is not trivially purely exogenous (i.e., the branching ratio n is not zero) and remains coupled to external influences, such as a continuous flow of noise fluctuations and sporadic large external events. First, let us consider a limiting case in which a system is purely exogenous ($n = 0$), and external events would trigger only localized reactions within the system, resulting in isolated exogenous shocks, while the rest of the system remains entirely static without any fluctuations. Naturally, such an idealized system does not

exist in reality. As long as $n > 0$, which is the general situation representing an internal organization of coupled subsystems, a large external event would have the tendency to trigger further events within the system, as we see in the exo-subcritical and exo-critical regimes. For example, in the exo-critical regime (Type II), the initial shock is exo, and it is followed by a potentially large cascade of triggered events expressing the endogenous dynamics. On the other hand, in the endo-critical regime (Type IV), the system is driven, as usual, by a myriad of small external noise fluctuations which are renormalized into cascades of endogenously triggered events. These triggered events can create spontaneously growing activity, culminating in an endo peak. The dynamics of activities before and after the endo peak are approximately symmetric, in contrast with the very strong asymmetry of an exo-critical shock. Here, the physics and dynamics are actually the same as for the exo cases (given $n > 0$), namely the internal driving mechanism is always the self-excitation and triggering within the system in the presence of small fluctuations ("noise"). Our model and classification framework acknowledge the intrinsic coupling between exogenous events and endogenous triggering processes. This should not be confused with our effort to distinguish endo and exo origins, which refers to the initial origin of a velocity peak, whether internal or external.

An entangled endo-exo scenario arises in the supercritical regime, where the system becomes progressively unstable and has a finite probability of experiencing exponential or super-exponential cascades of events. It is important to note that the driving mechanism remains the same as in the subcritical and critical regimes, with the system's response still governed by self-excitation and triggering. This supercritical regime reflects a state of strong mutual interactions among the system's constituents, where clear cause-effect relationships break down. The system becomes highly endogenous, fragile, and extremely sensitive to external perturbations. The perturbation from an external event, even if minor, can be drastically amplified by endogenous positive feedbacks, explaining the ambiguous cause-effect relationship between the early May 2012 rainfall and the eventual collapse of the Preonzo slope roughly 10 days later. It is important to clarify that this does not imply a more dominant exogenous component; rather, it is the endogenous component that becomes more influential, with the transition to a branching ratio $n > 1$ driving an explosive branching process and intense cascade of disturbances. Thus, it is the endogenous processes that amplify the impact of an exogenous event, making the exogenous effect appear more prominent.

It is worth noting subtle differences in the endo-exo origin of system responses between the subcritical/critical regimes, where an endo-exo classification is feasible, and the supercritical regime, where such classification becomes challenging. For instance, in exo-critical landslide dynamics, a system response initiated by an exogenous trigger is endogenously amplified through cascades of mass interactions; however, the origin of this chain reaction is clearly exo. In other words, if there is no exo trigger, there is no shock and no associated recovery. This is different from the entangled endo-exo scenario in the supercritical regime. For the 2012 failure event of the Preonzo slope, if there were no rainfall in early May 2012, the slope might still fail eventually, but the rainfall made the failure to occur earlier. Thus, classifying the initial trigger of the final failure as endogenous or exogenous becomes both difficult and, ultimately, irrelevant. Once the system has entered an unstable regime, any of a wide range of perturbations can be amplified. The proximate exogenous trigger is secondary to the underlying structural instability. It is akin to attributing the fall of a pen balanced on your finger to a passing breeze or a slight hand movement. The key point is that the system is inherently unstable and primed to amplify

disturbances. The fall itself is merely a symptom of this intrinsic fragility, not the result of a singular cause.

Text S3: Stochastic theory of landslide triggering cascades

The results in the Main Text have been obtained by using the first-order moment (or average) of the landslide mass velocity, i.e., Eq. 1. We can improve the theory by accounting for the fact that the full process is stochastic with each mother mass motion potentially triggering a number of daughter mass motions according to the productivity law of mass movement triggering:

$$\rho(E) = \rho_0 (E/E_0)^a, \tag{S1}$$

defining the average number of daughter masses triggered by a mother mass of energy release $E \geq E_0$, where E_0 is an energy cutoff (i.e., a mass with energy release below E_0 does not trigger daughter masses), and ρ_0 and a are positive constants.

Drawing parallels between landslides and earthquakes (*7*, *10*, *15*), we postulate that the probability density function of daily energy release of a landslide follows a Gutenberg-Richter-type law as:

$$f(E(t)) \propto E(t)^{-(1+\mu)}, \tag{S2}$$

where μ is a positive exponent. Given $E(t) \propto v(t)^2$ and by applying the law of conservation of probability under a change of variable (*33*), we obtain the probability density function of daily velocities as:

$$f(v(t)) \propto v(t)^{-(1+2\mu)}. \tag{S3}$$

Since the probability distribution of the $v(t)$'s of the Preonzo landslide follows an inverse gamma distribution (with β denoting its shape parameter; see text S4) characterized by a power law tail $f(v(t)) \propto v(t)^{-(1+\beta)}$, we therefore obtain:

$$\beta = 2\mu. \tag{S4}$$

Given $\mu = 2b/3$ and the stress-dependence of the seismic b-value (*55*), we may expect $b \approx 1.2 - 1.4$, $\mu \approx 0.80 - 0.93$, and therefore $\beta \approx 1.60 - 1.87$ for landslides (given the differential stress being about a few MPa (*69*)). This is consistent with the β value we obtain in the current study for the Preonzo landslide in the supercritical regime (see Fig. 7D and fig. S11B) as well as the β values of two other landslides during their crises obtained in our previous study (*44*).

The branching ratio n is nothing but the average productivity and given by (*57*):

$$n = \int_{\tau}^{+\infty} \int_{E_0}^{+\infty} f(E)\rho(E)\psi(t-\tau)\,dE\,dt. \tag{S5}$$

For $\vartheta > 0$ and $a < \mu$, given Eq. 7 in Materials and Methods, the double integral in Eq. S5 leads to a finite value for n (*57*):

$$n = \rho_0\mu/(\mu - a) = \rho_0\beta/(\beta - 2a). \tag{S6}$$

Strictly speaking, Eq. S6 is valid only in the subcritical and critical regimes with $n \leq 1$. However, it can still be used for $n > 1$, albeit with caution, by interpreting values of $n > 1$ as indicative of a system in a critical or transiently supercritical destabilizing regime.

From Eq. 9 in Materials and Methods, we obtain, for the subcritical regime:

$$n = \left[1 + \Gamma(1-\vartheta)(c/t^*)^{\vartheta}\right]^{-1}. \tag{S7}$$

By combing Eqs. S6 and S7, we obtain:

$$\rho_0 = (1 - 2a/\beta)\left[1 + \Gamma(1 - \vartheta)(c/t^*)^{\vartheta}\right]^{-1}. \quad \text{(S8)}$$

For the Type I exogenous-subcritical peak observed at the Preonzo landslide (Fig. 5A and fig. S6A), we have the characteristic time $t^* \approx 8$ days. According to the literature of earthquake aftershocks (*25*, *29*), c may vary from days to minutes. Here, we estimate $c \approx 1$ day based on the power law decay trends of the Preonzo landslide around peaks, while a smaller c (e.g., 0.1 day) tends to overestimate n (fig. S11B). Furthermore, we postulate $a \approx 0.8$, which is the typical value for earthquakes (*70*) and proven to also hold for the Preonzo landslide (see below). Given $\vartheta \approx 0.45$, using Eq. S7, we obtain $n \approx 0.61$ for this exogenous-subcritical peak. Based on the profile maximum likelihood estimation (*47*), we find $\beta \approx 2.0$ during the relaxation stage of this peak (fig. S11A). By using Eq. S8, we further constrain $\rho_0 \approx 0.12$. Finally, based on the time series of the β value (fig. S11A) determined by fitting the velocity data to the inverse gamma distribution (text S4), we obtain an estimation of the temporal evolution of n (fig. S11B).

Note that the calculation of n here is intended only as a first-order approximation, where local fluctuations are generally smoothed out in deriving β from the probability density function of daily velocities. This explains the appearance of some exogeneous/endogenous-critical peaks when n is not so close to 1 (compare figs. S11B and S12). A more accurate estimation of n may be achieved by calibrating the underlying self-exciting Hawkes point process to the velocity time series using the maximum likelihood estimation method (*71*), to be explored in future work.

Since the total number of daughter masses triggered by a mother event of energy E per day scales as $\rho(E)f(E) \propto E^{a-\mu}$ (*70*), we expect that the transition from the subcritical/critical regime (for which $a - \mu < 0$) to the supercritical regime (for which $a - \mu > 0$) is characterized by a shift from $a \lesssim \mu = \beta/2$ to $a > \mu = \beta/2$. Given β progressively drops from 1.82 to 1.62 over 1-2 months prior to the final collapse (see Fig. 7D), we expect $a \approx 0.8 - 0.9$, which is consistent with our postulation above and comparable to the typical value of $a \approx 0.8$ for earthquakes (*70*). This correspondence holds notwithstanding the fact that landslides happen in near-surface environments under low stress conditions, while earthquakes occur in deep subsurface regions subject to much higher stress levels. Our results demonstrating parallels between landslides and earthquakes provide additional supports for the fault mechanics perspective of landslide dynamics and failure (*7*, *10*, *15*). It is worth noting that some distinct characteristics may exist between landslides and earthquakes. For example, the relationship between the ϑ value in the subcritical/critical regime and the p value in the supercritical regime derived in the previous research in the context of earthquake aftershocks (*34*) does not explain our results for the Preonzo landslide. Further investigation is needed to better understand this.

<u>Text S4: Inverse gamma distribution</u>

The probability density function of the three-parameter inverse gamma distribution is written as (*47*):

$$f(v) = \frac{\alpha^{\beta}}{\Gamma(\beta)}\left(\frac{1}{v-\gamma}\right)^{\beta+1} \exp\left(-\frac{\alpha}{v-\gamma}\right), \quad \text{(S9)}$$

where v is the slope velocity, α is a scale parameter, β is a shape parameter equal to the exponent of the asymptotic power law tail for large v's (according to the mathematical convention in the theory of Lévy stable laws (*33*)), γ is a threshold velocity, and $\Gamma(\cdot)$ is the gamma function. The parameters need to meet the conditions of $\alpha > 0$, $\beta > 0$, and $\gamma < v$ for $f(v)$ to qualify as a normalized probability density function. The parameters α, β, and γ can be determined based on the profile maximum likelihood estimation method (*47*). The inverse

gamma distribution has an essential singularity at $v = \gamma$ with a rollover for v's around the mode $\alpha/(\beta + 1) + \gamma$, and a power law decay with a tail exponent β for medium and large v's, so that the tail of the inverse gamma is described by the power law (*44*):

$$f(v) \approx \frac{\alpha^{\beta}}{\Gamma(\beta)} v^{-\beta-1}, \text{ for } v \gg \alpha + \gamma. \tag{S10}$$

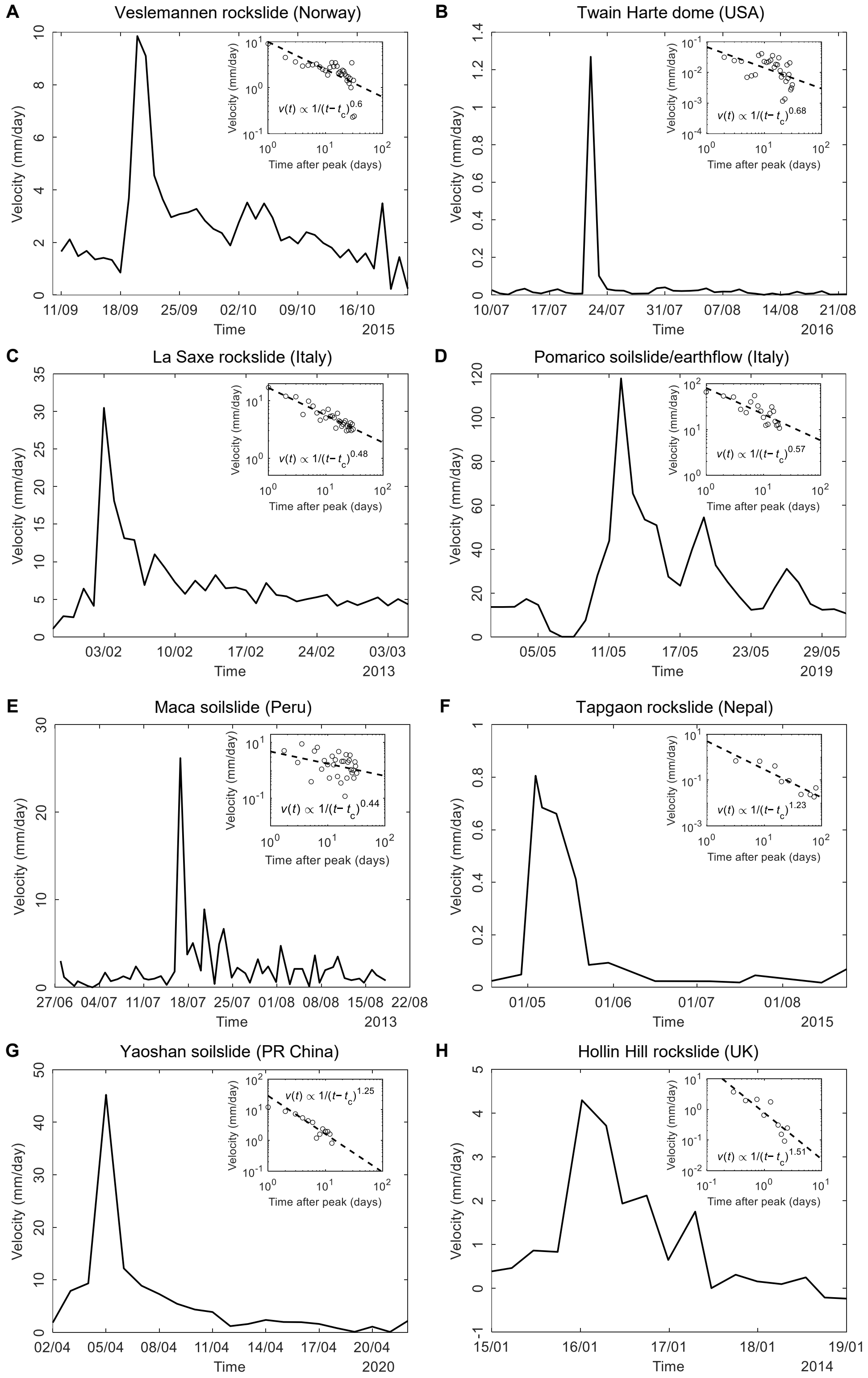

Fig. S1.

Exogenous peaks observed in various landslides. Insets qualify the power law singularity dynamics of the velocity $v(t)$ as a function of time after the peak at time t_c.

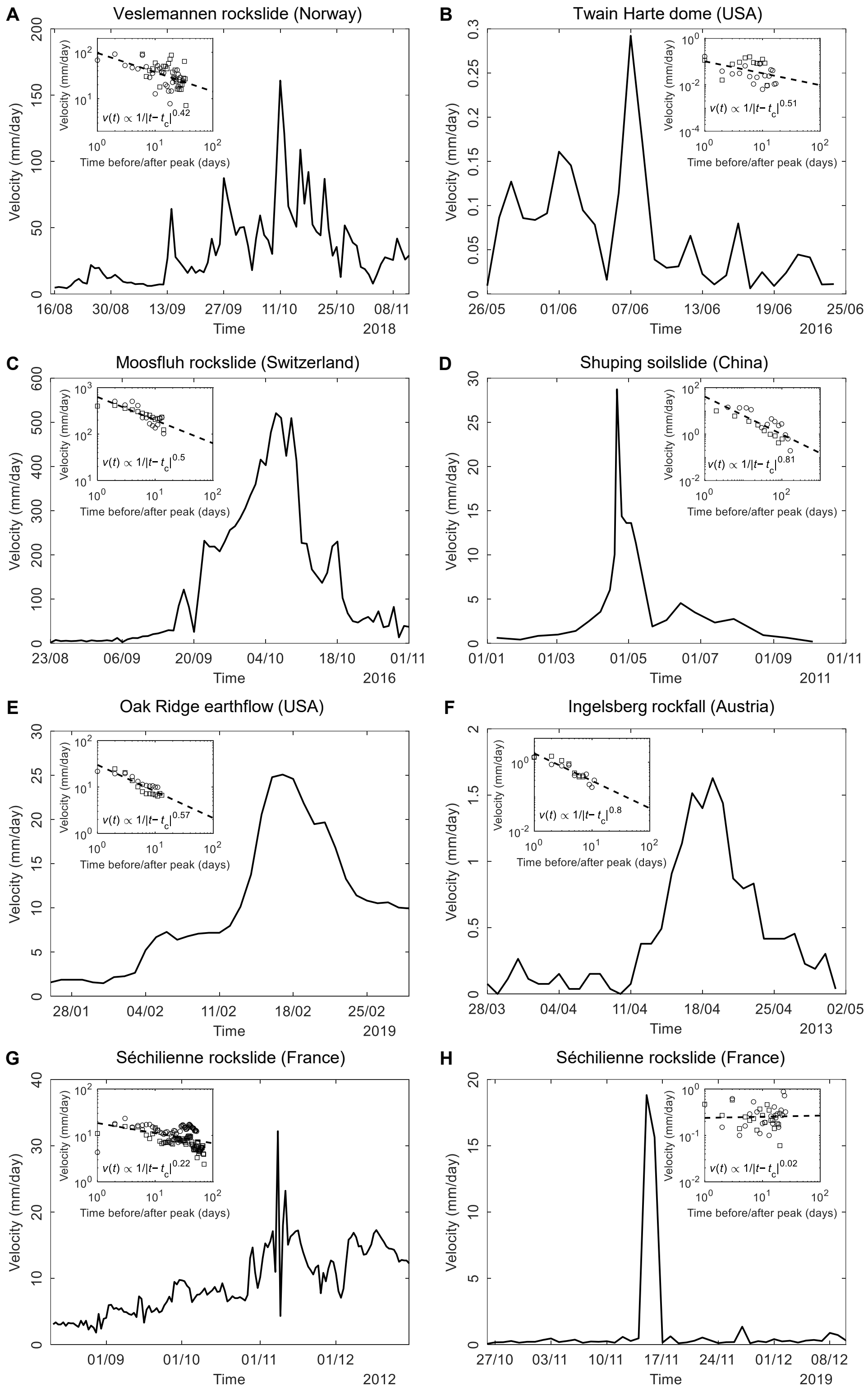

Fig. S2.

Endogenous peaks observed in various landslides. Insets qualify the power law singularity dynamics of the velocity $v(t)$ as a function of time before (squares) and after (circles) the peak at time t_c.

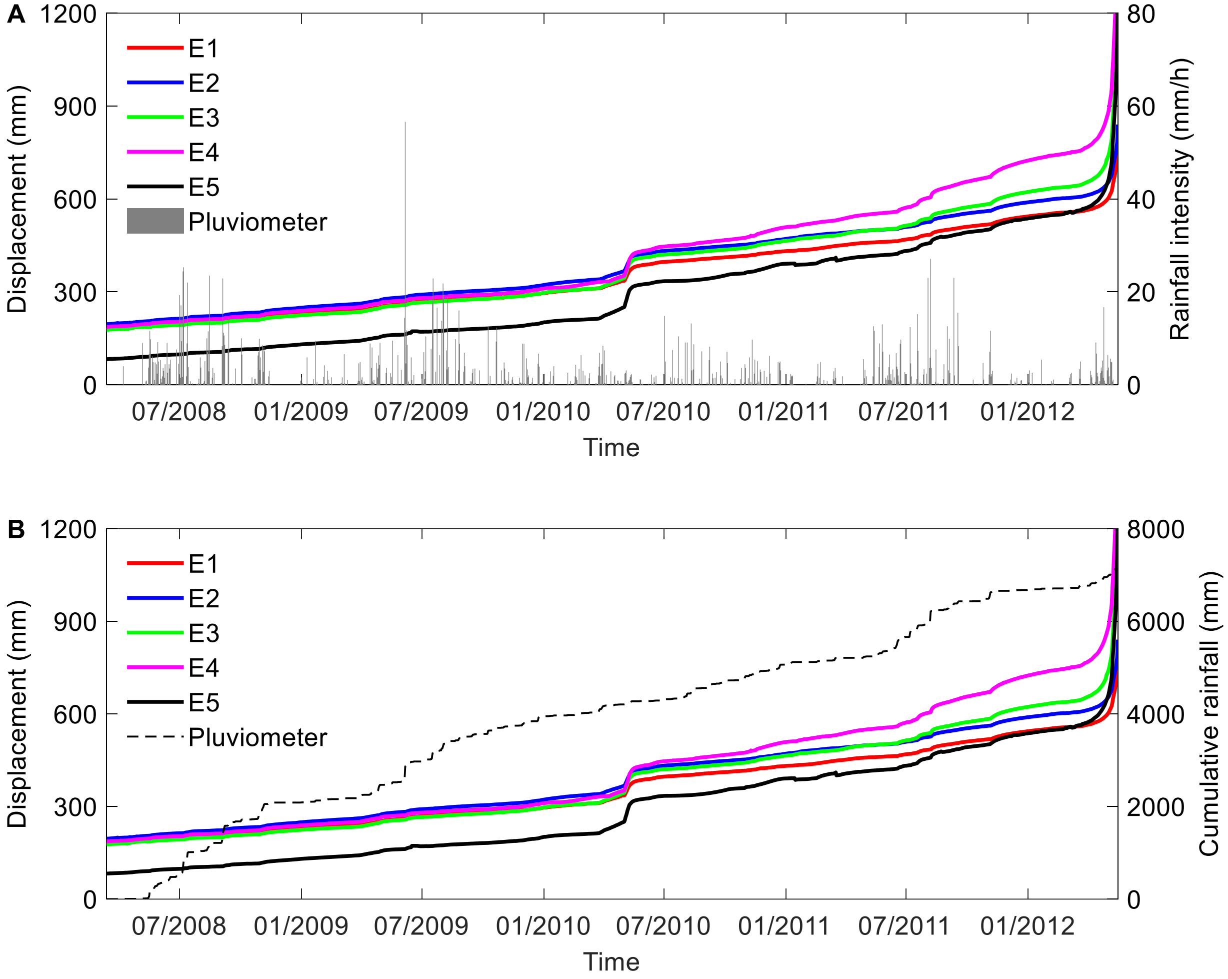

Fig. S3.

Monitoring data of the Preonzo landslide, Switzerland. Time series of slope displacements measured by five extensometers presented together with the data of (**A**) rainfall intensity and (**B**) cumulative rainfall amount recorded by a pluviometer installed at the Preonzo slope.

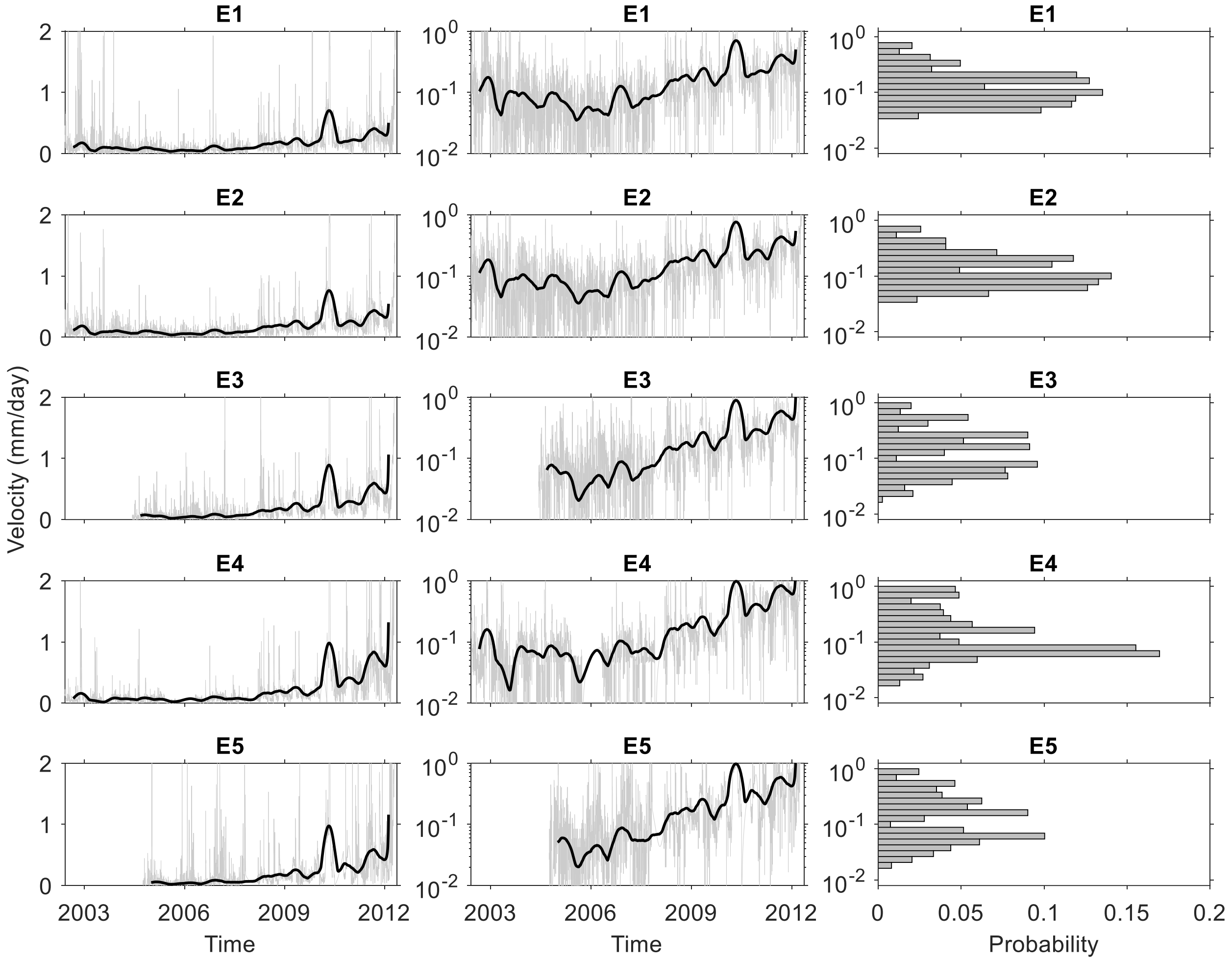

Fig. S4.

Long-term velocity trends of the Preonzo landslide. In the left and middle panels, velocities are plotted on the linear and logarithmic scales, respectively; the gray line gives the nonsmoothed velocity time series and the black line gives the smoothed velocity by the Savitzky-Golay filter with a polynomial order of 2 and a frame length of 6 months. The right panel gives histograms approximating the probability density function of long-term velocities.

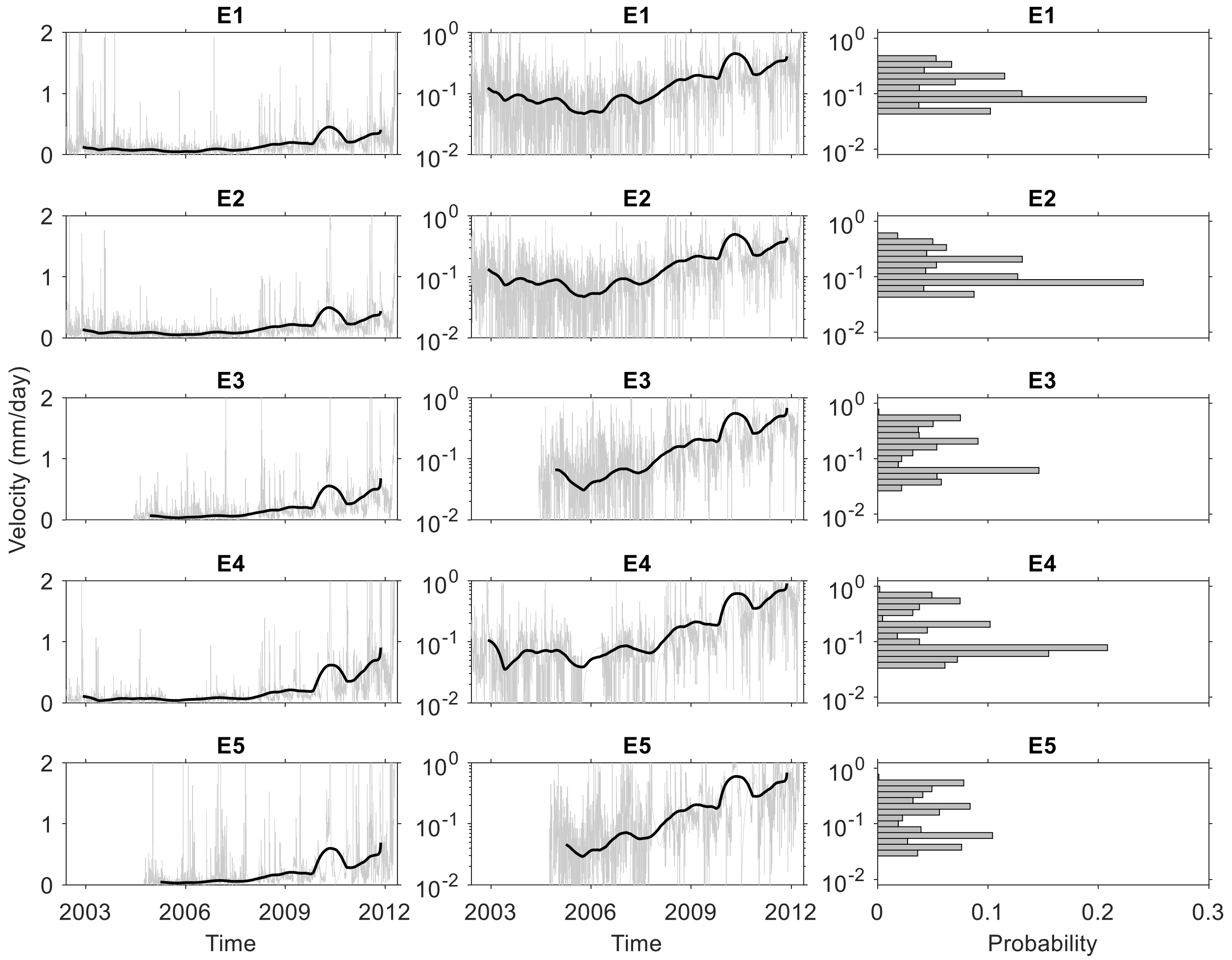

Fig. S5.

Long-term velocity trends of the Preonzo landslide. In the left and middle panels, velocities are plotted on the linear and logarithmic scales, respectively; the gray line gives the nonsmoothed velocity time series and the black line gives the smoothed velocity by the Savitzky-Golay filter with a polynomial order of 2 and a frame length of 1 year. The right panel gives histograms approximating the probability density function of long-term velocities.

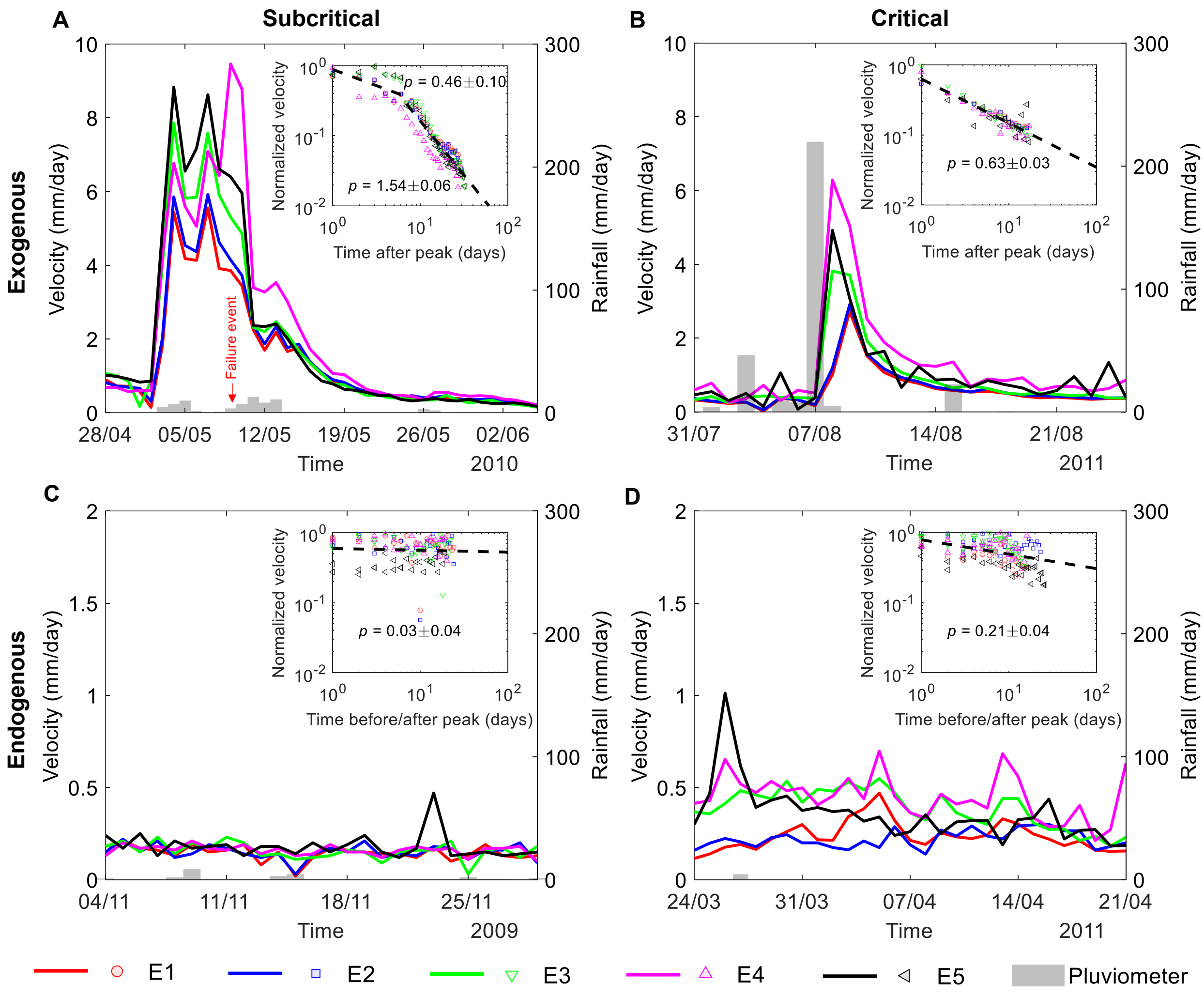

Fig. S6.

Time series of daily slope velocity measured by the five extensometers E1-E5 and time series of rainfall data recorded by the pluviometer for different types of peaks (insets show the post-peak velocity relaxation). (**A**) Type I, exogenous-subcritical; (**B**) Type II, exogenous-critical; (**C**) Type III, endogenous-subcritical; and (**D**) Type IV, endogenous-critical. The red arrow in **A** marks the timing of the local failure of a northern sector of the slope on 9 May 2010. For endogenous peaks as shown in **C** and **D**, pre-peak velocity data are also indicated (open markers) in addition to post-peak data (filled markers).

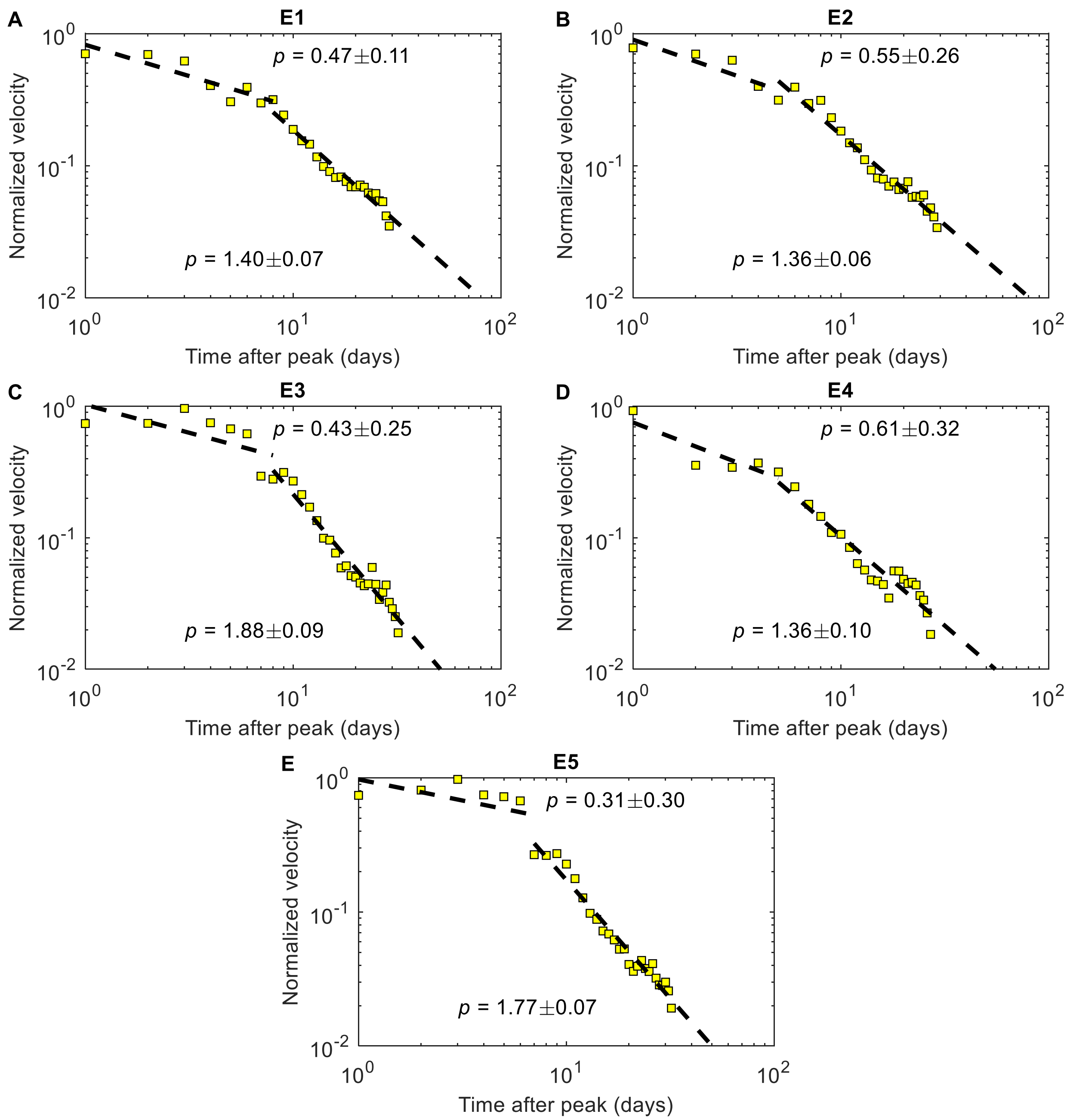

Fig. S7.

Post-peak relaxation of Type I exogenous-subcritical peaks. Variation of normalized velocity as a function of post-peak time for the five extensometers E1-E5.

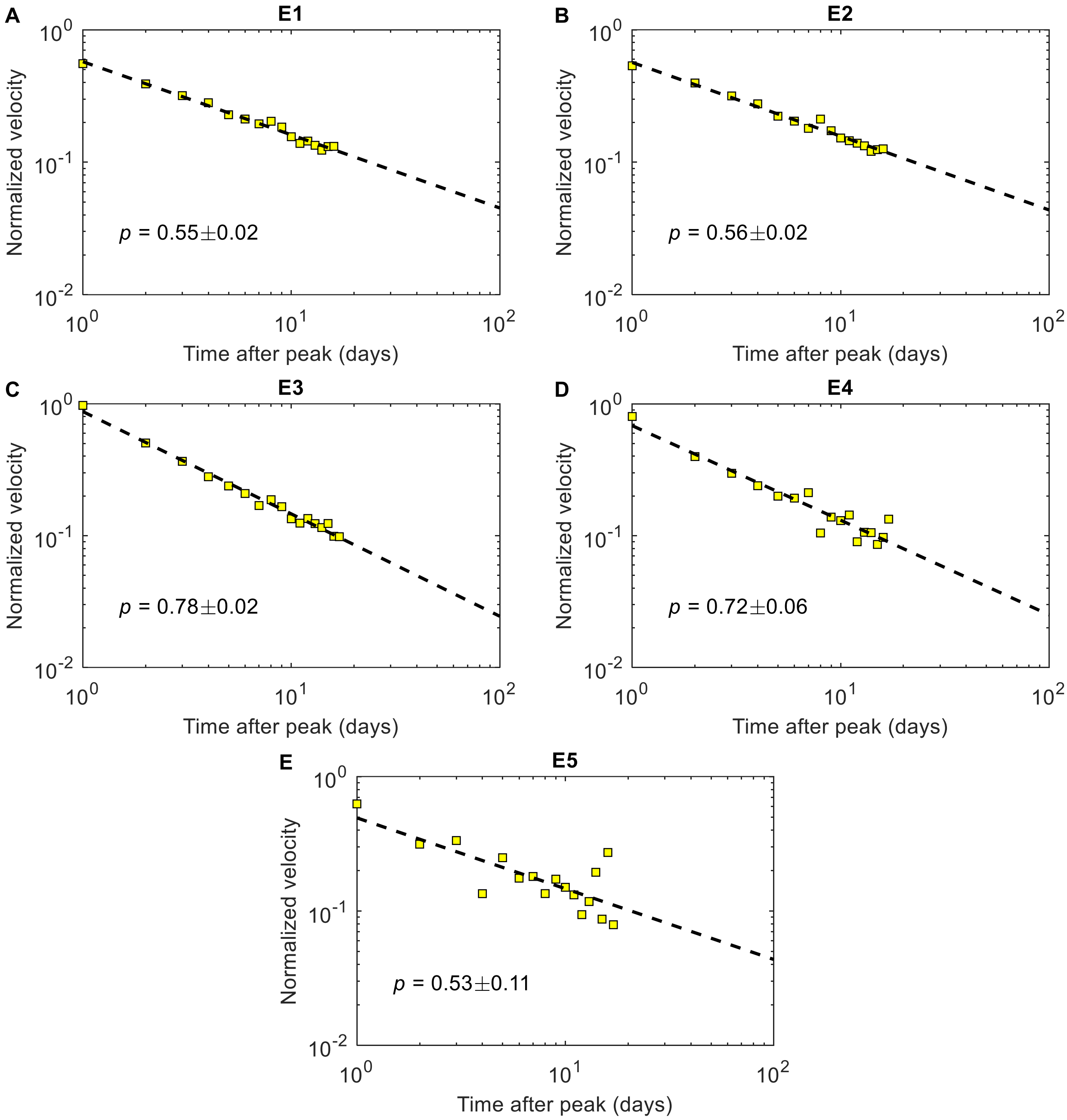

Fig. S8.

Post-peak relaxation of Type II exogenous-critical peaks. Variation of normalized velocity as a function of post-peak time for the five extensometers E1-E5.

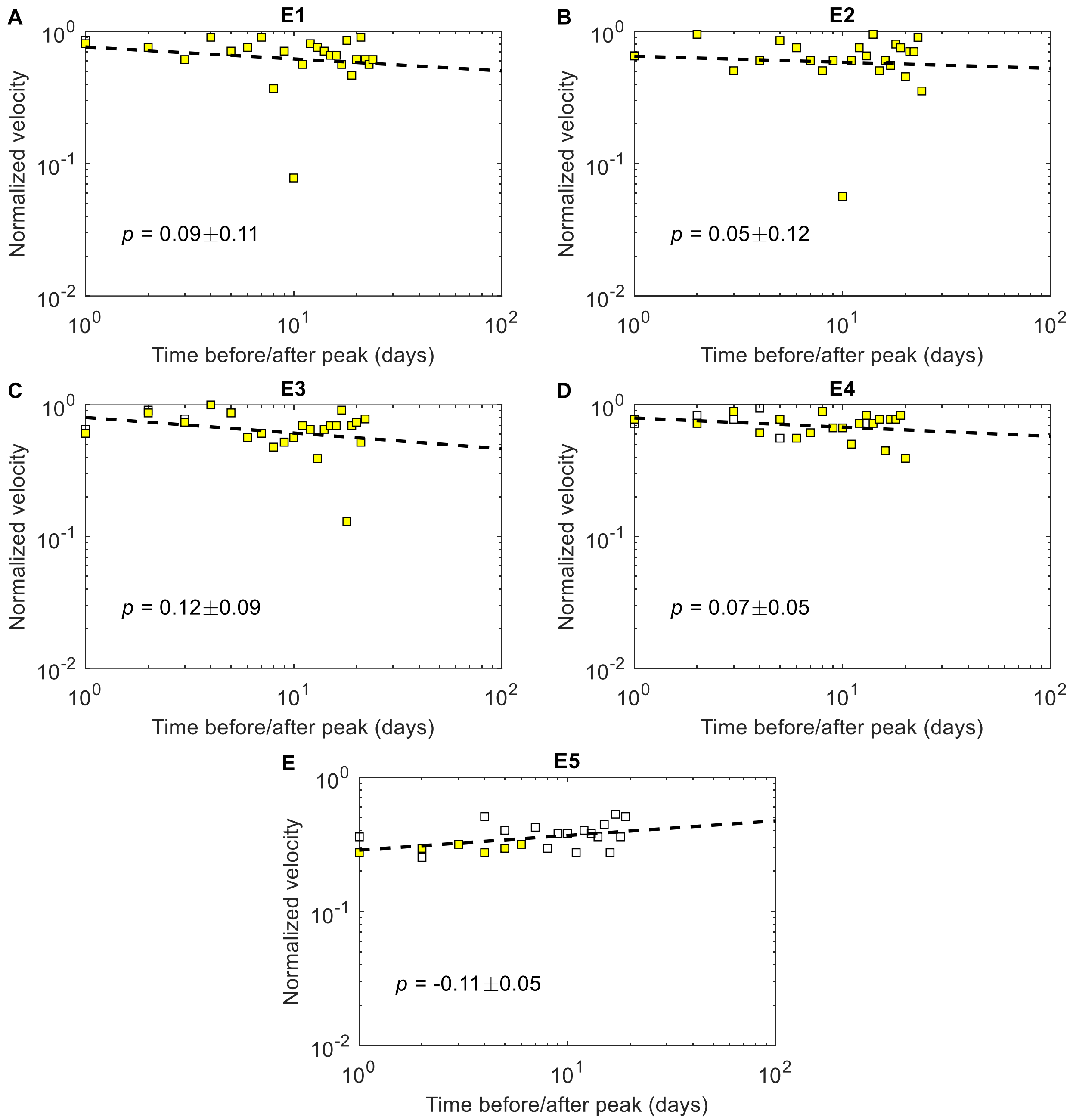

Fig. S9.

Pre-peak (open symbols) acceleration and post-peak (colored symbols) relaxation of Type III endogenous-subcritical peaks. Variation of normalized velocity as a function of pre/post-peak time for the five extensometers E1-E5.

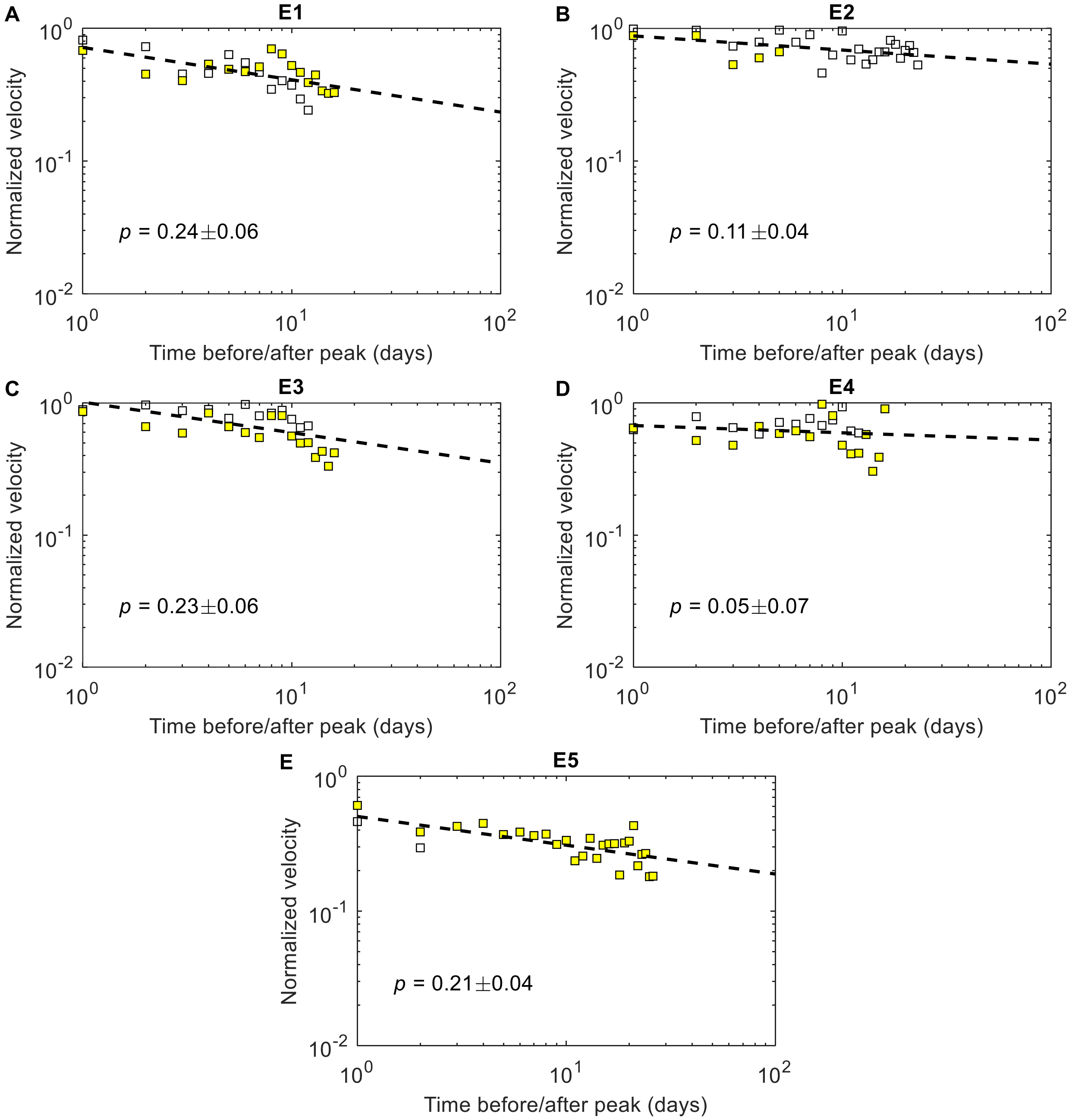

Fig. S10.

Pre-peak (open symbols) acceleration and post-peak (colored symbols) relaxation of Type IV endogenous-critical peaks. Variation of normalized velocity as a function of pre/post-peak time for the five extensometers E1-E5.

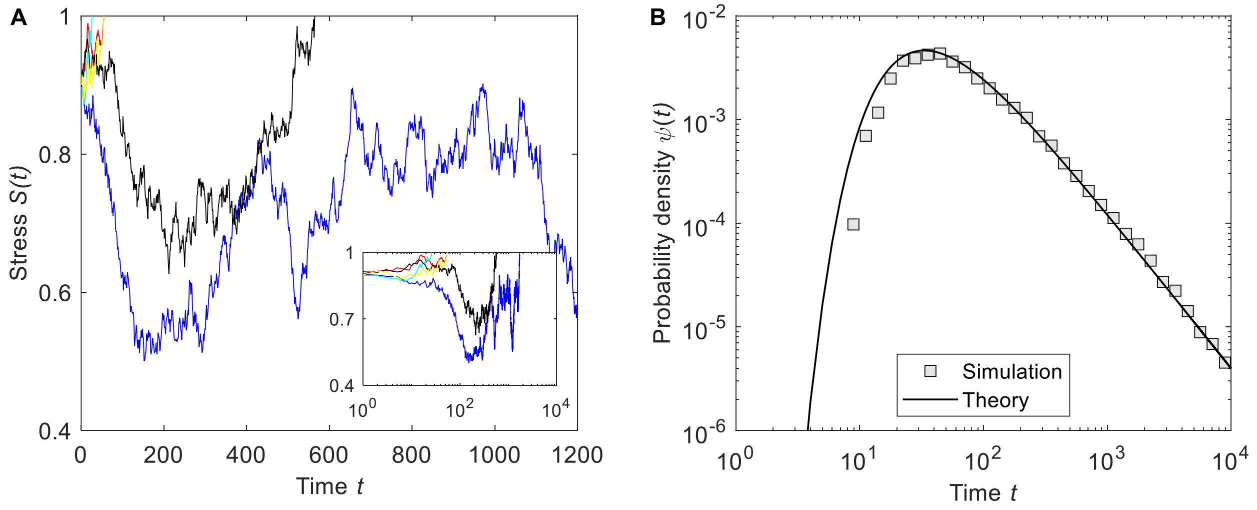

Fig. S11.

Random walk simulation of stress fluctuations with a comparison to the theory of first-passage processes. The random walk, with each step taking a unit time, is associated with a diffusion coefficient of $D = 1 \times 10^{-4}$. The waiting time corresponds to when the stress $S(t)$ with an initial value of $S_0 = 0.9$ for the first time exceeds the critical value of $S_c = 1.0$. (**A**) Stress history of 5 realizations (selected arbitrarily from 10000 realizations); inset shows the plot with time in log-log scale. (**B**) Probability density function of waiting times obtained from the random walk simulation with a comparison to the theoretical solution of the first-passage problem: $\psi(t) = (S_c - S_0)(2\pi D t^3)^{-1/2}\exp[-(S_c - S_0)^2/(2Dt)]$, which asymptotically converges to a power law $\psi(t) \propto t^{-3/2}$.

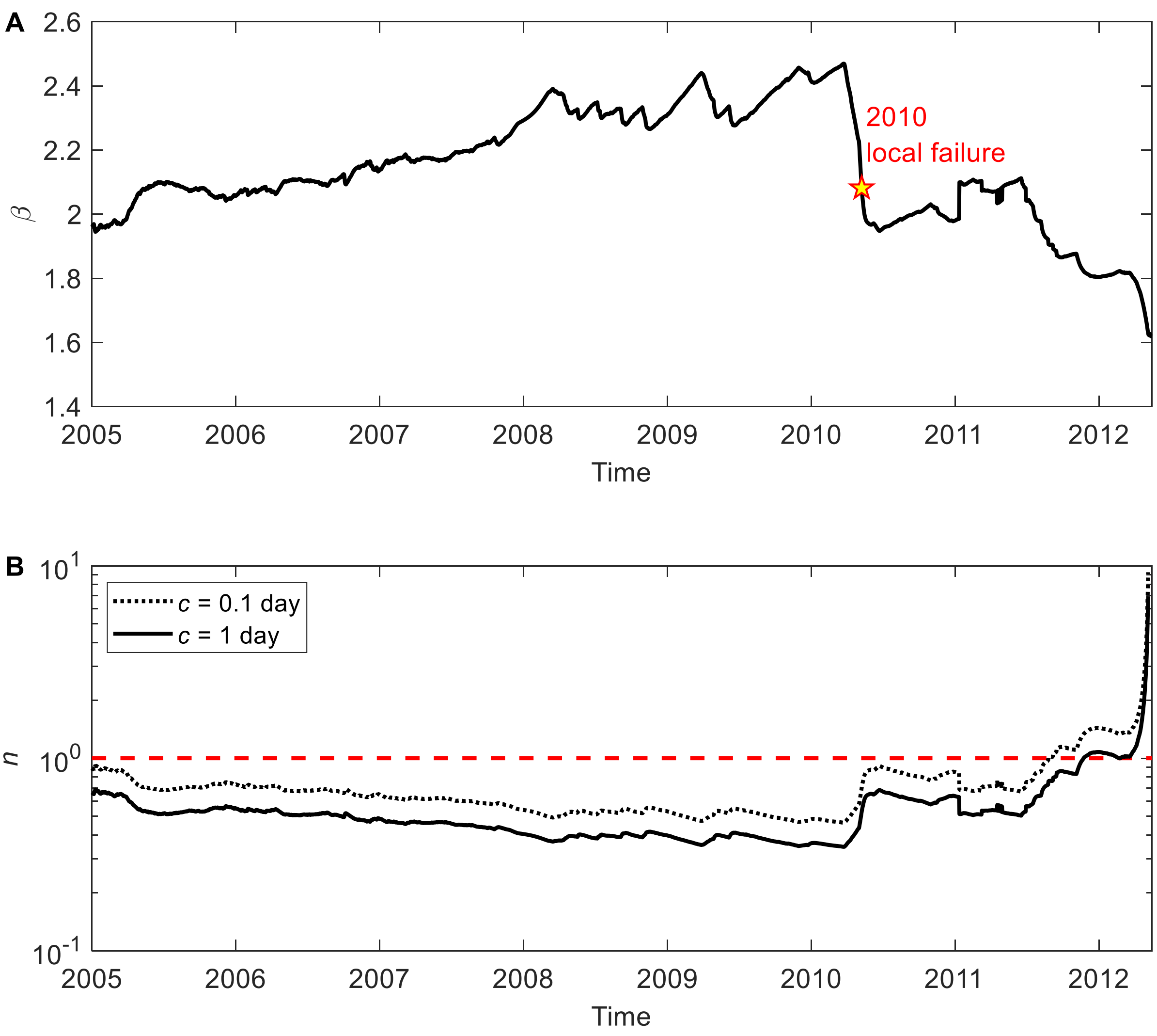

Fig. S12.

Temporal variation of the β-value and n-value of the Preonzo landslide. (**A**) Temporal evolution of the power law tail exponent β of the velocity probability distribution estimated based on the profile maximum likelihood estimation method; only the β evolution after 2005 is shown because sufficient data are needed to constrain the velocity probability distribution; the pentagram marker indicates the 2010 local failure event, prior to which a notable decline of the β-value can also be observed. (**B**) Temporal evolution of the branching ratio n (see text S3 for the calibration procedures). The dashed red horizonal line in **B** marks the critical condition of $n = 1$.

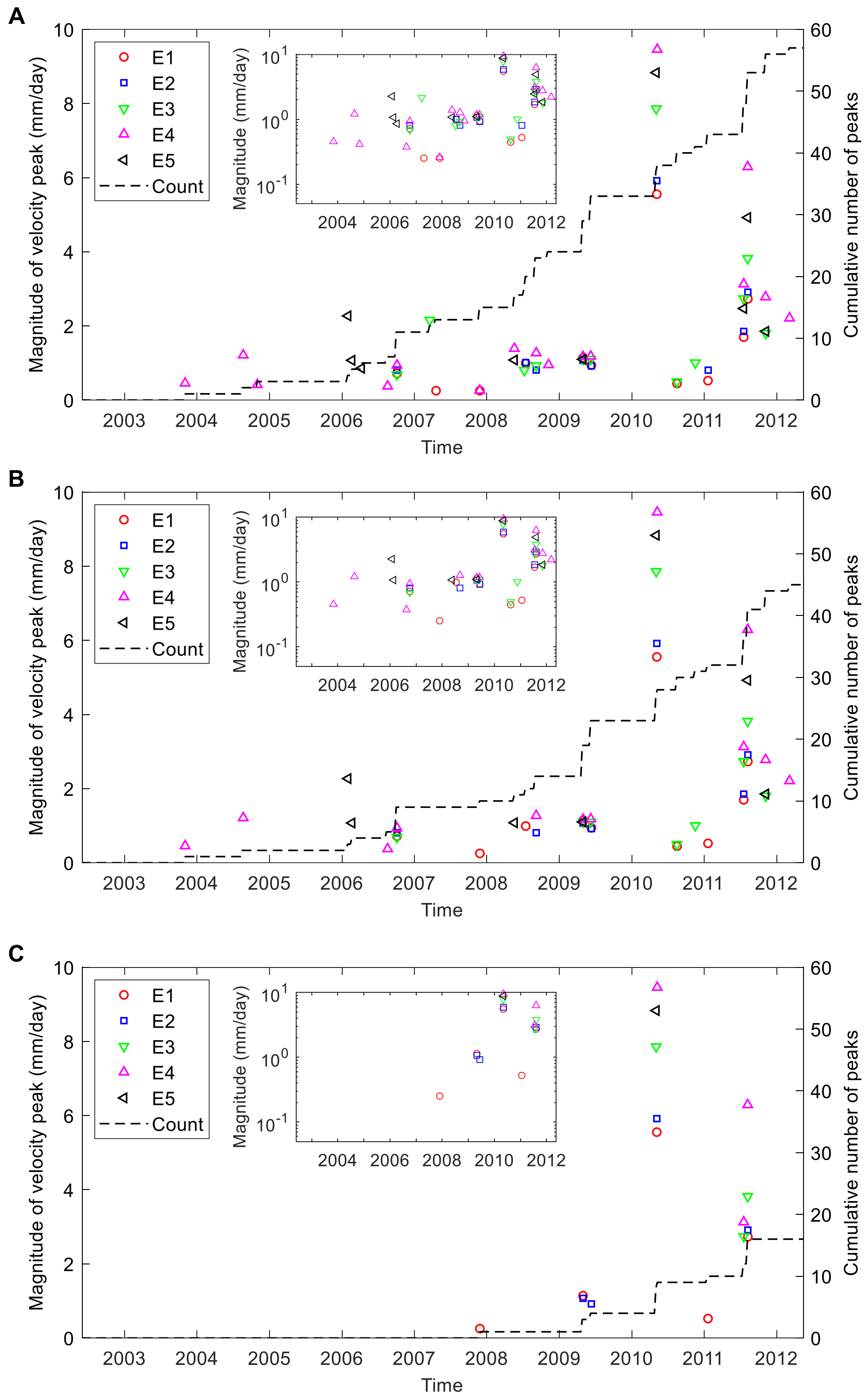

Fig. S13.

Temporal evolution of the magnitude and cumulative number of exogenous velocity peaks. The peaks are selected from those shown in fig. S17 with an additional criterion that the coefficient of determination (in the power law fitting) (**A**) $R^2 > 0.7$ (see fig. S22A), (**B**) $R^2 > 0.8$ (see Fig. 6), and (**C**) $R^2 > 0.9$ (see fig. S22B).

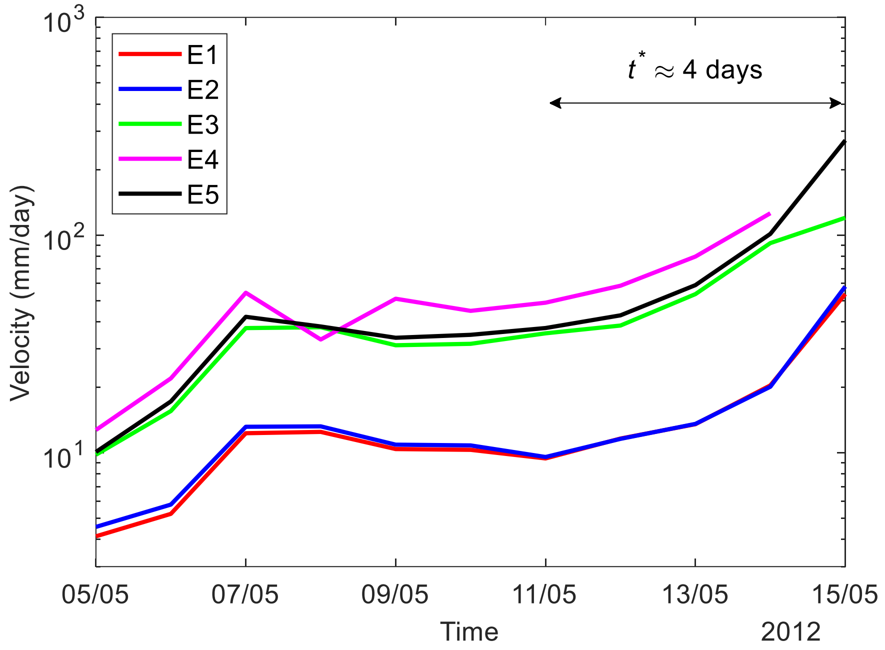

Fig. S14.

Precursory deceleration of the Preonzo landslide prior to its catastrophic failure on 15 May 2012. Here, the characteristic time t^* marks the transition from an early-time deceleration (from 7 to 11 May) to a late-time acceleration (from 11 May to 15 May).

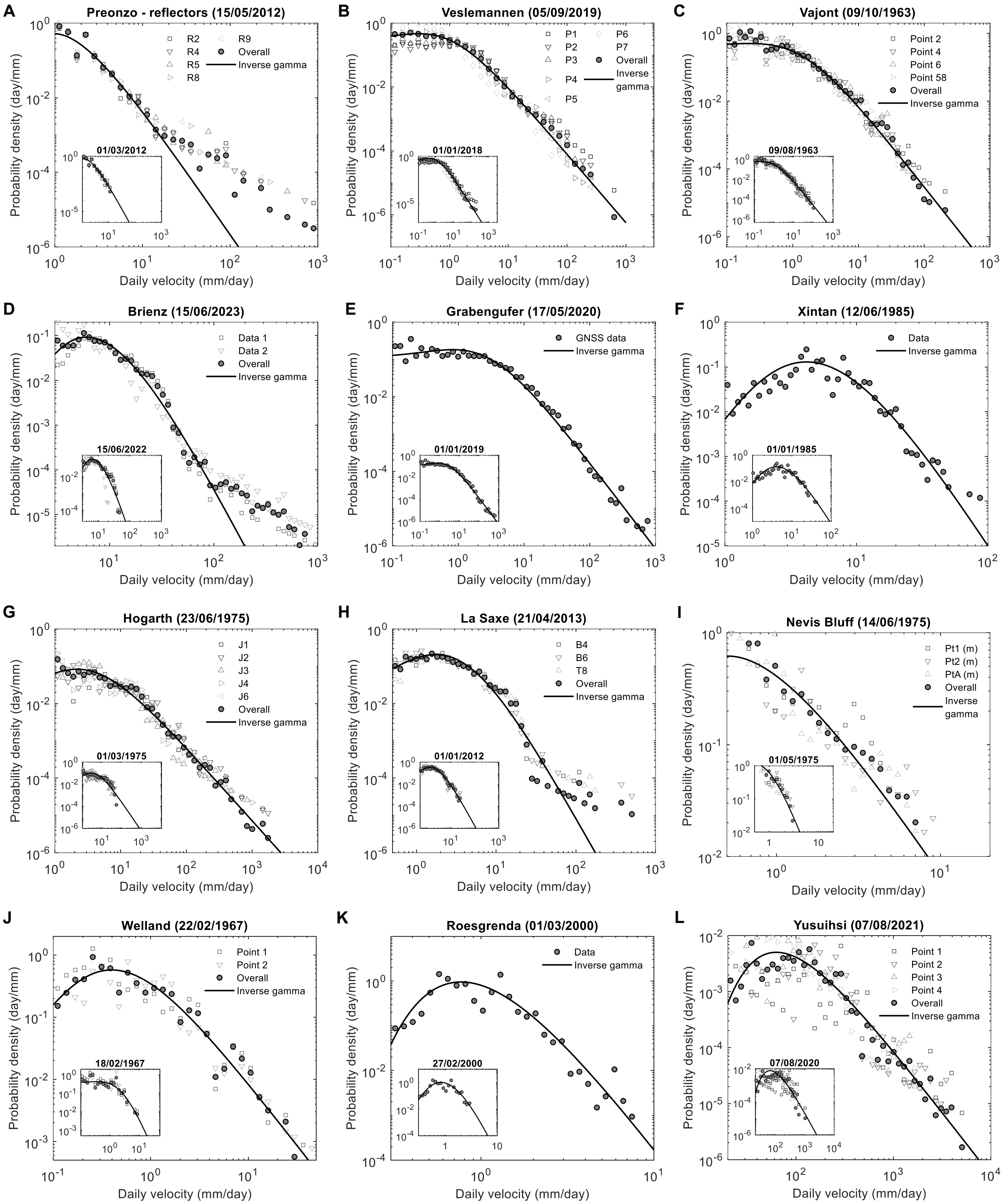

Fig. S15.

Probability density function of slope velocities fitted to the inverse gamma function for various landslides.

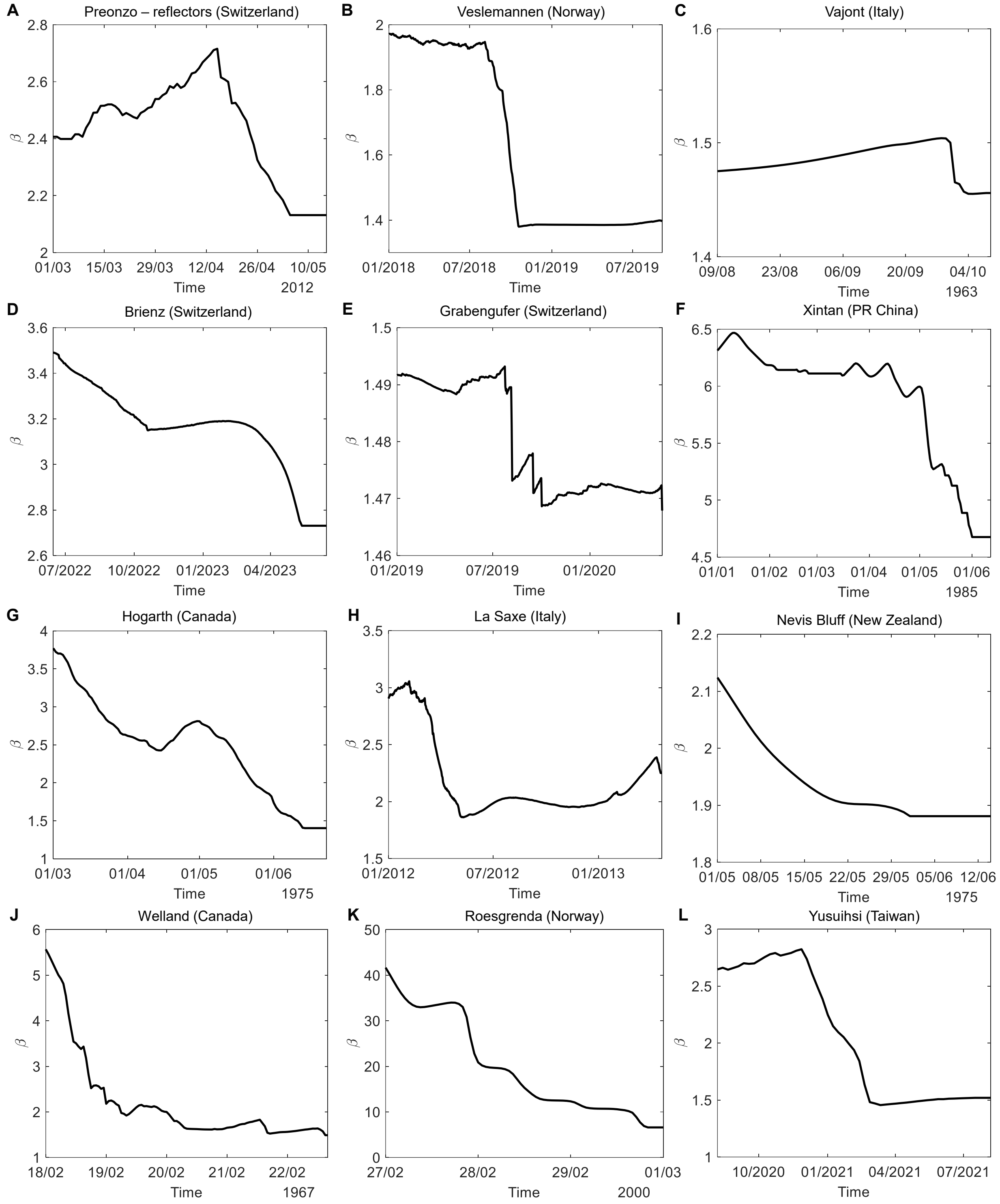

Fig. S16.

Progressive decrease with time of exponent *β* of the slope velocity probability distribution preceding catastrophic failure in various landslides.

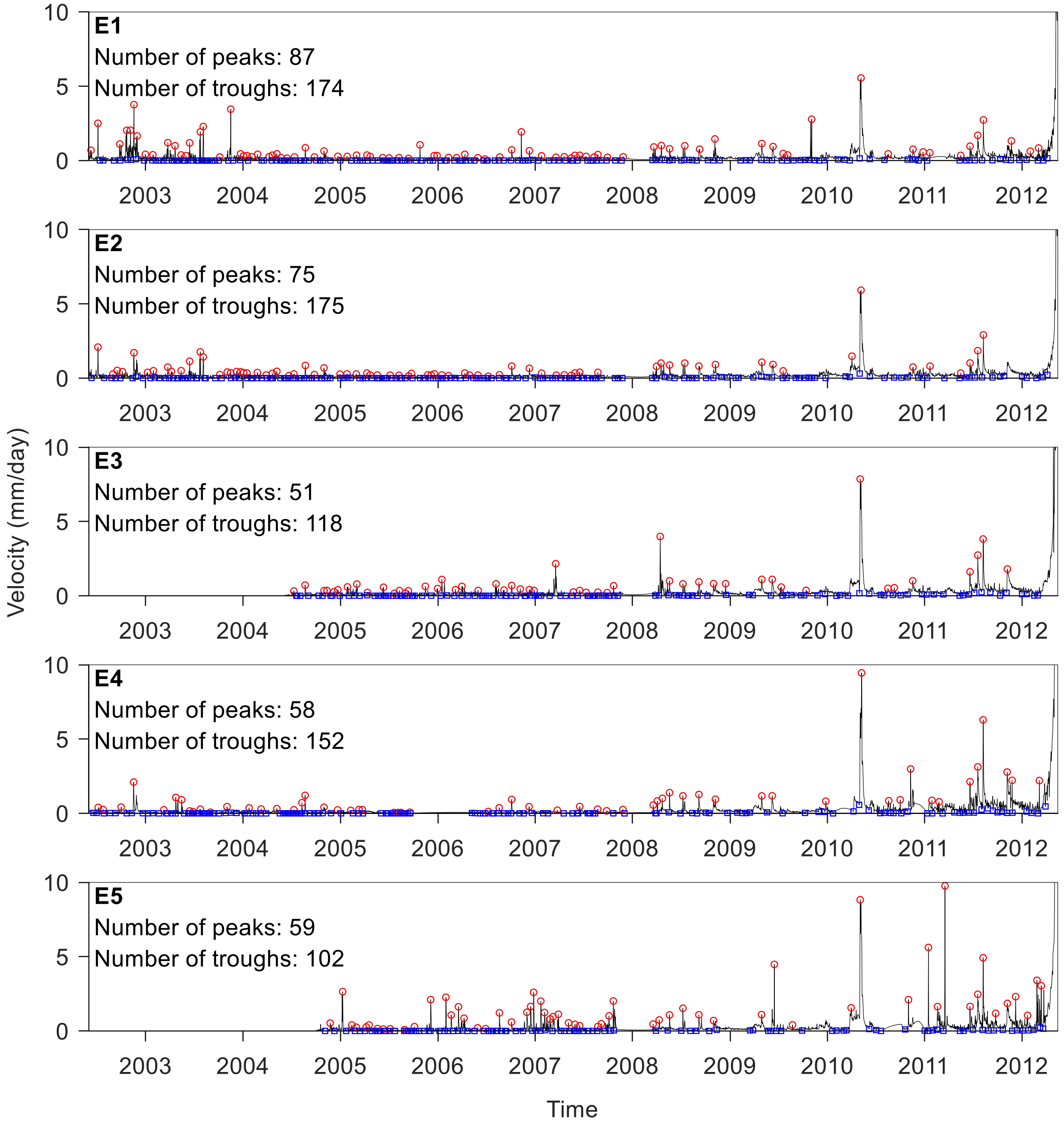

Fig. S17.

Time series of daily slope velocities recorded by the five extensometers E1-E5 (from top to bottom) instrumented at the Preonzo landslide, Switzerland. Peaks and troughs are marked by red circles and blue squares, respectively. Each peak (respectively trough) is qualified as a local maximum (respectively minimum) over a 20-day time window which is at least $k = 2.5$ times larger (respectively smaller) than the average velocity over a 2-month time window.

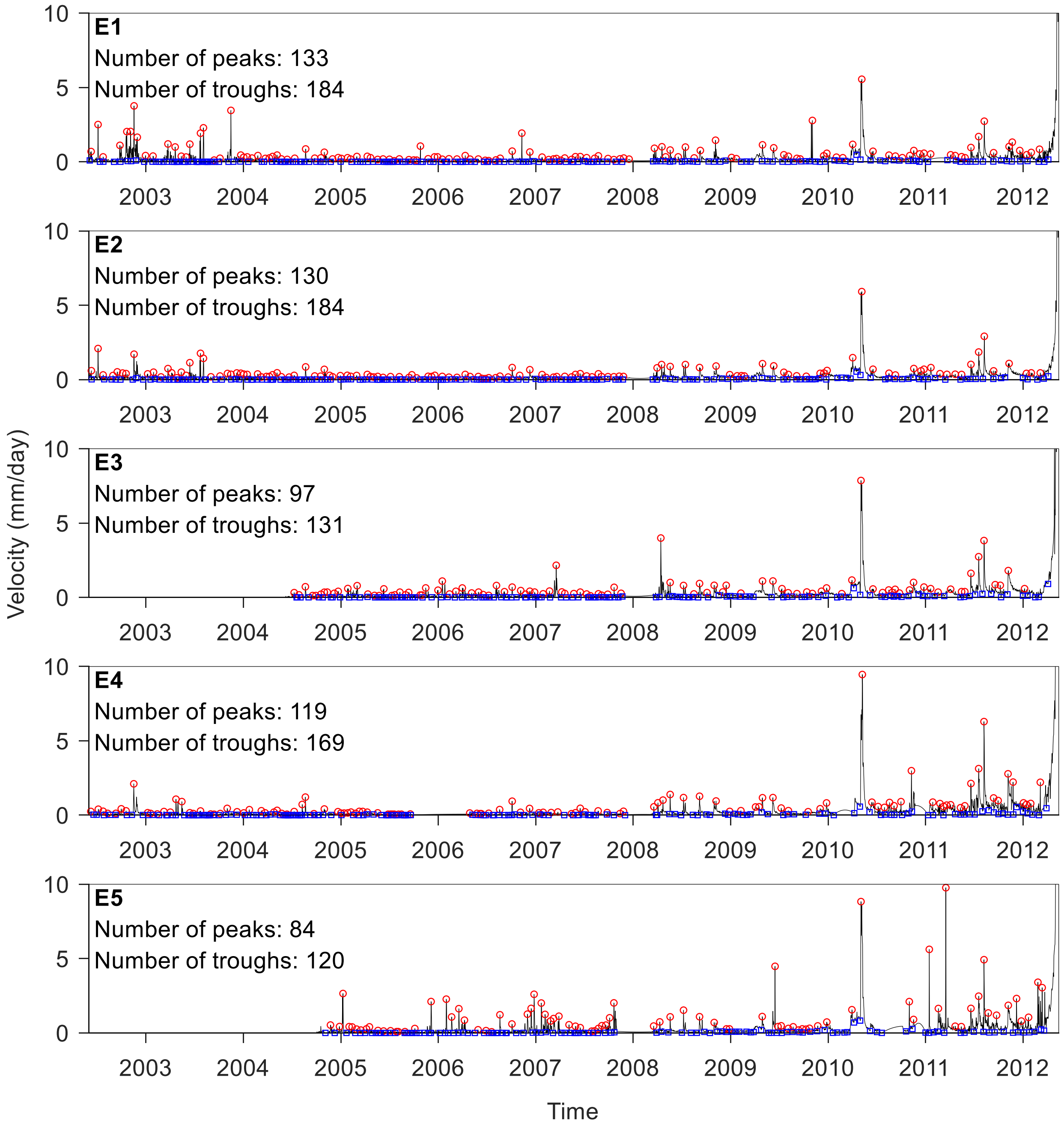

Fig. S18.

Time series of daily slope velocities recorded by the five extensometers E1-E5 (from top to bottom) instrumented at the Preonzo landslide, Switzerland. Peaks and troughs are marked by red circles and blue squares, respectively. Each peak (respectively trough) is qualified as a local maximum (respectively minimum) over a 20-day time window which is at least $k = 1.5$ times larger (respectively smaller) than the average velocity over a 2-month time window.

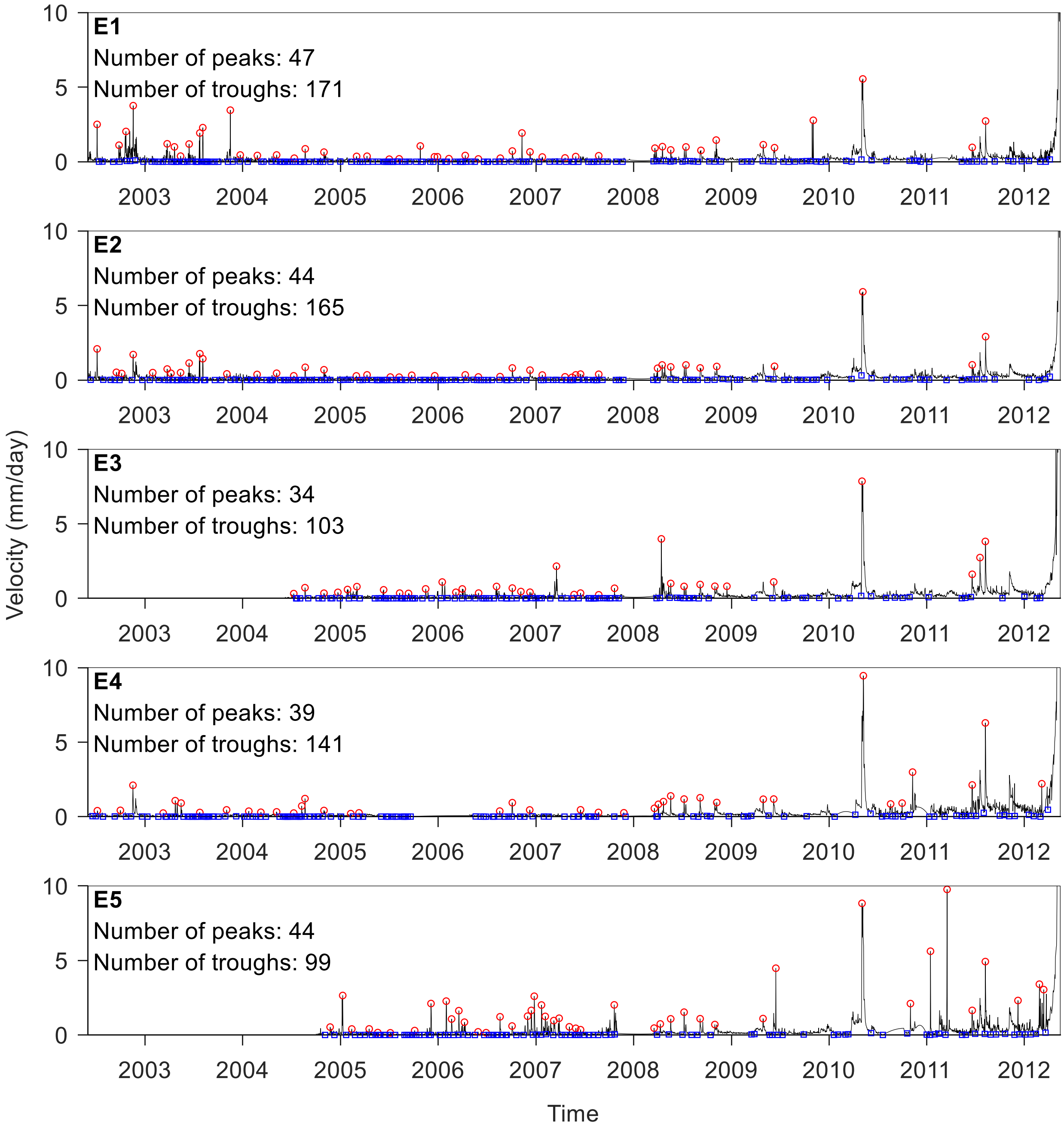

Fig. S19.

Time series of daily slope velocities recorded by the five extensometers E1-E5 (from top to bottom) instrumented at the Preonzo landslide, Switzerland. Peaks and troughs are marked by red circles and blue squares, respectively. Each peak (respectively trough) is qualified as a local maximum (respectively minimum) over a 20-day time window which is at least $k = 3.5$ times larger (respectively smaller) than the average velocity over a 2-month time window.

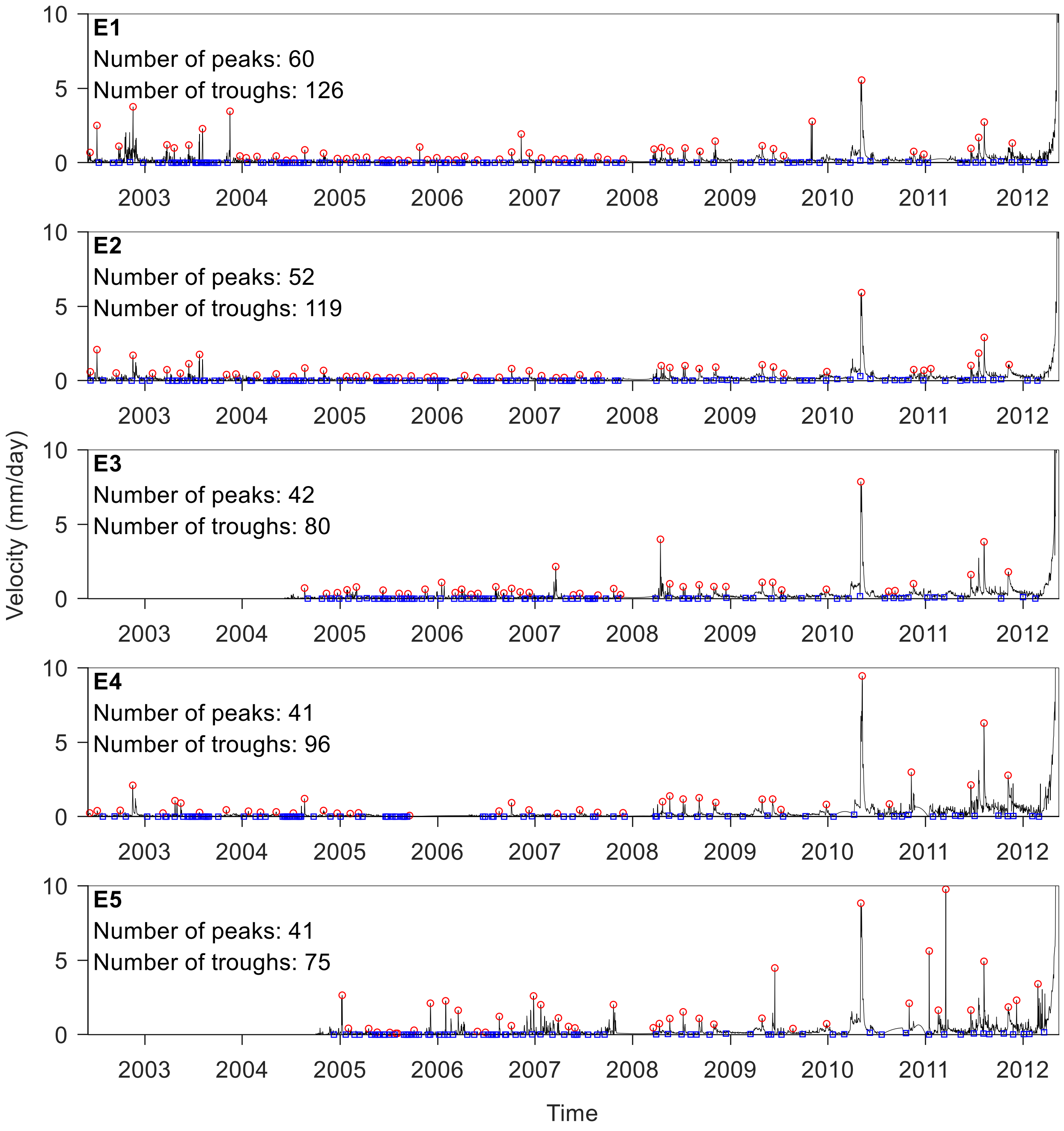

Fig. S20.

Time series of daily slope velocities recorded by the five extensometers E1-E5 (from top to bottom) instrumented at the Preonzo landslide, Switzerland. Peaks and troughs are marked by red circles and blue squares, respectively. Each peak (respectively trough) is qualified as a local maximum (respectively minimum) over a 40-day time window which is at least $k = 2.5$ times larger (respectively smaller) than the average velocity over a 4-month time window.

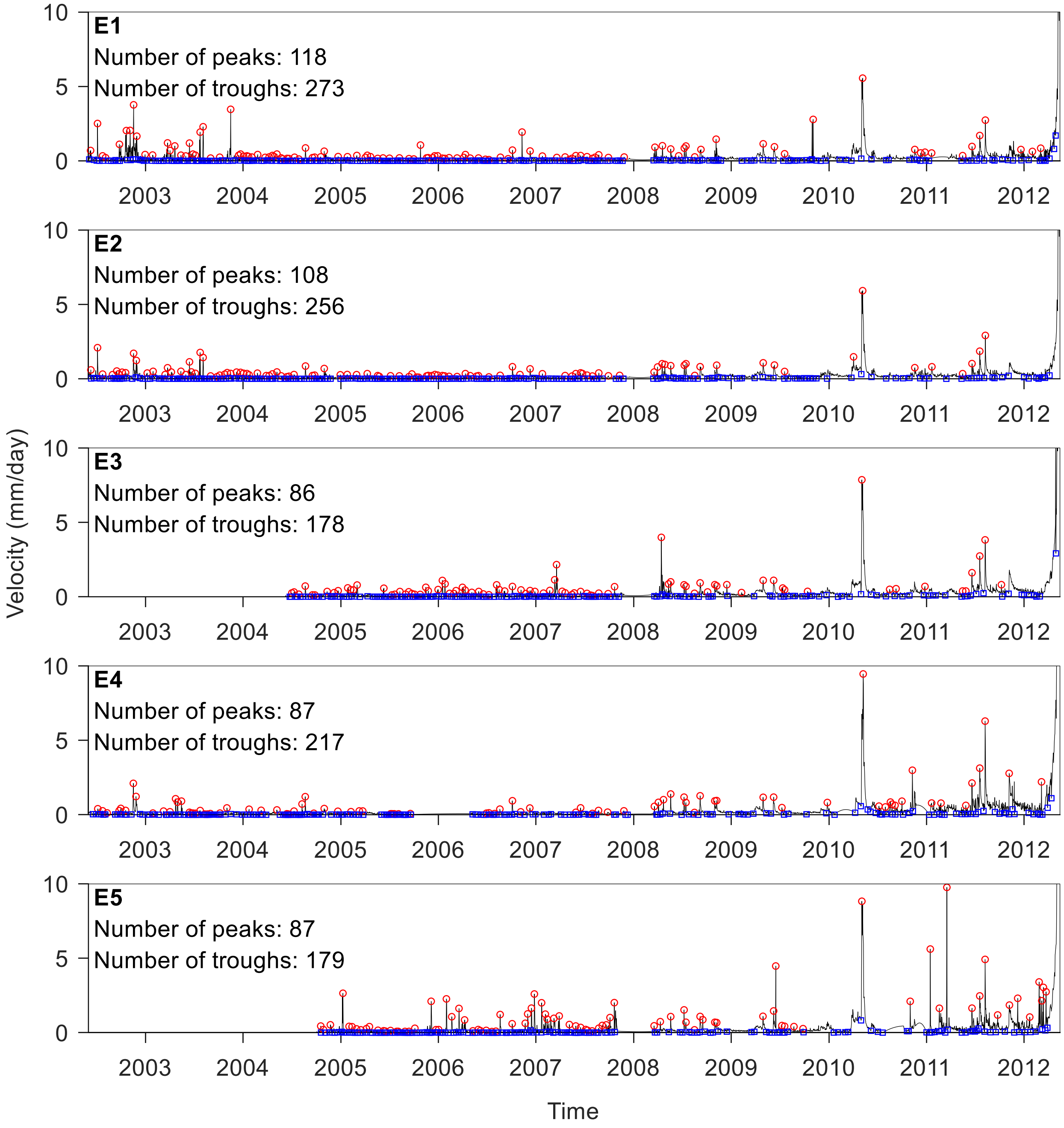

Fig. S21.

Time series of daily slope velocities recorded by the five extensometers E1-E5 (from top to bottom) instrumented at the Preonzo landslide, Switzerland. Peaks and troughs are marked by red circles and blue squares, respectively. Each peak (respectively trough) is qualified as a local maximum (respectively minimum) over a 10-day time window which is at least $k = 2.5$ times larger (respectively smaller) than the average velocity over a 1-month time window.

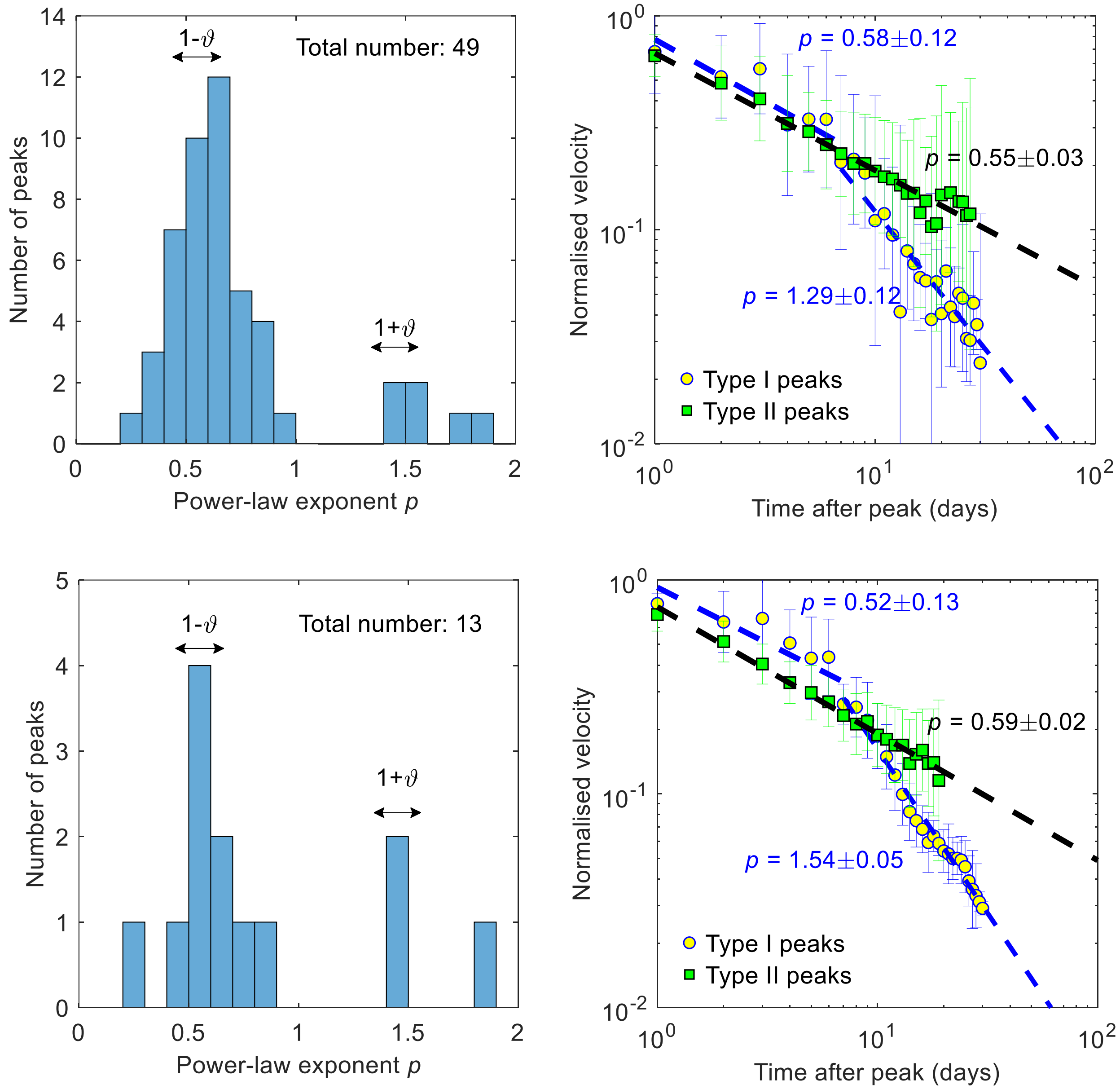

Fig. S22.

Post-peak relaxation properties associated with detected peaks in the velocity time series. Left: histogram of the power law exponents *p* for post-peak velocity relaxation. Right: ensemble averaged relaxation of Type I (exogenous-subcritical) and Type II (exogenous-critical) peaks. Here, a peak is qualified as a local maximum over a 20-day time window which is at least $k = 2.5$ times larger than the average velocity over a 2-month time window, while the coefficient of determination for the fitting should meet $R^2 > 0.7$ (upper panel) or $R^2 > 0.9$ (lower panel).

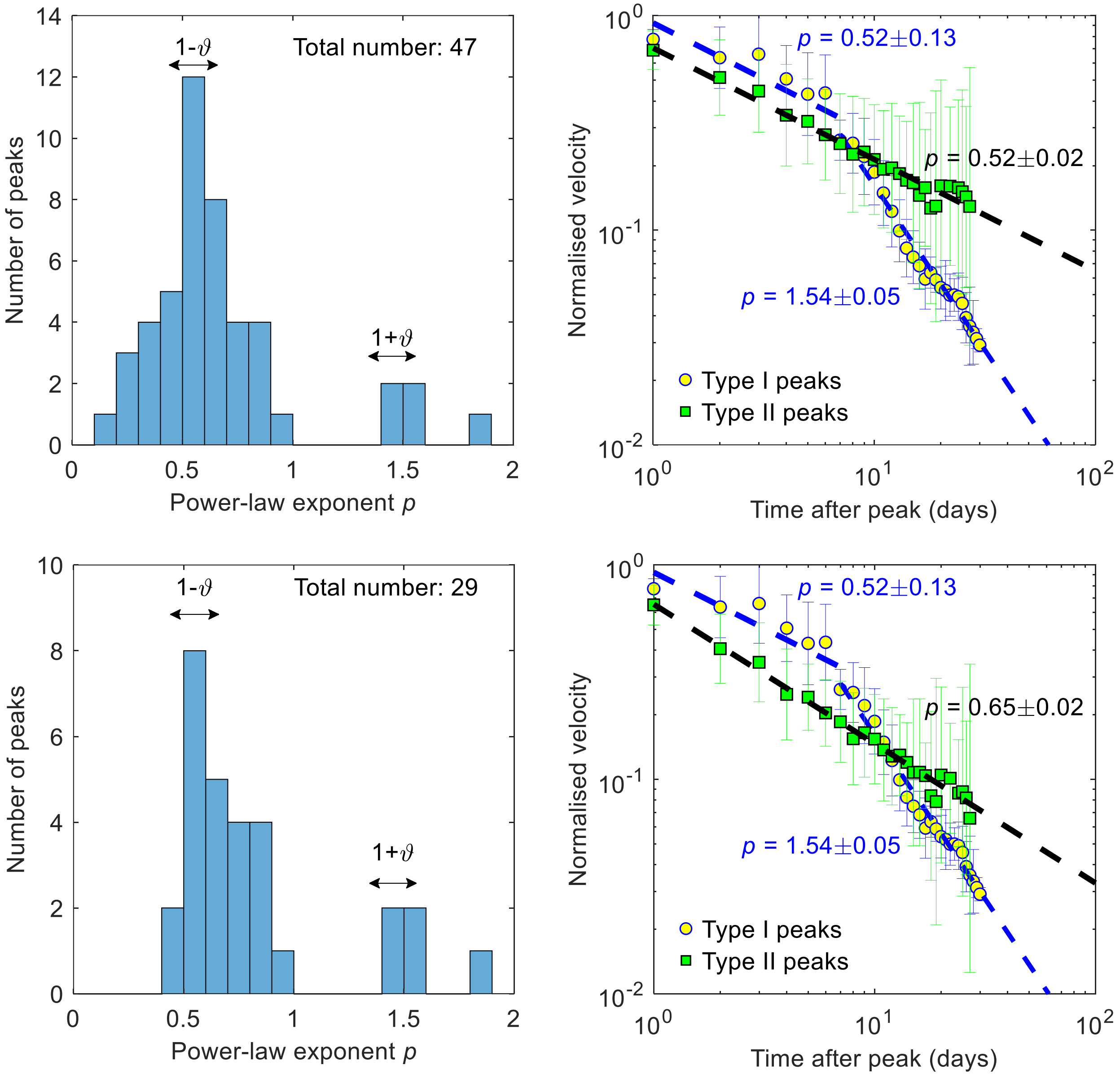

Fig. S23.

Post-peak relaxation properties associated with detected peaks in the velocity time series. Left: histogram of the power law exponents *p* for post-peak velocity relaxation. Right: ensemble averaged relaxation of Type I (exogenous-subcritical) and Type II (exogenous-critical) peaks. Here, a peak is qualified as a local maximum over a 20-day time window which is at least $k = 1.5$ (upper panel) or $k = 3.5$ (lower panel) times larger than the average velocity over a 2-month time window, while the coefficient of determination for the fitting should meet $R^2 > 0.8$.

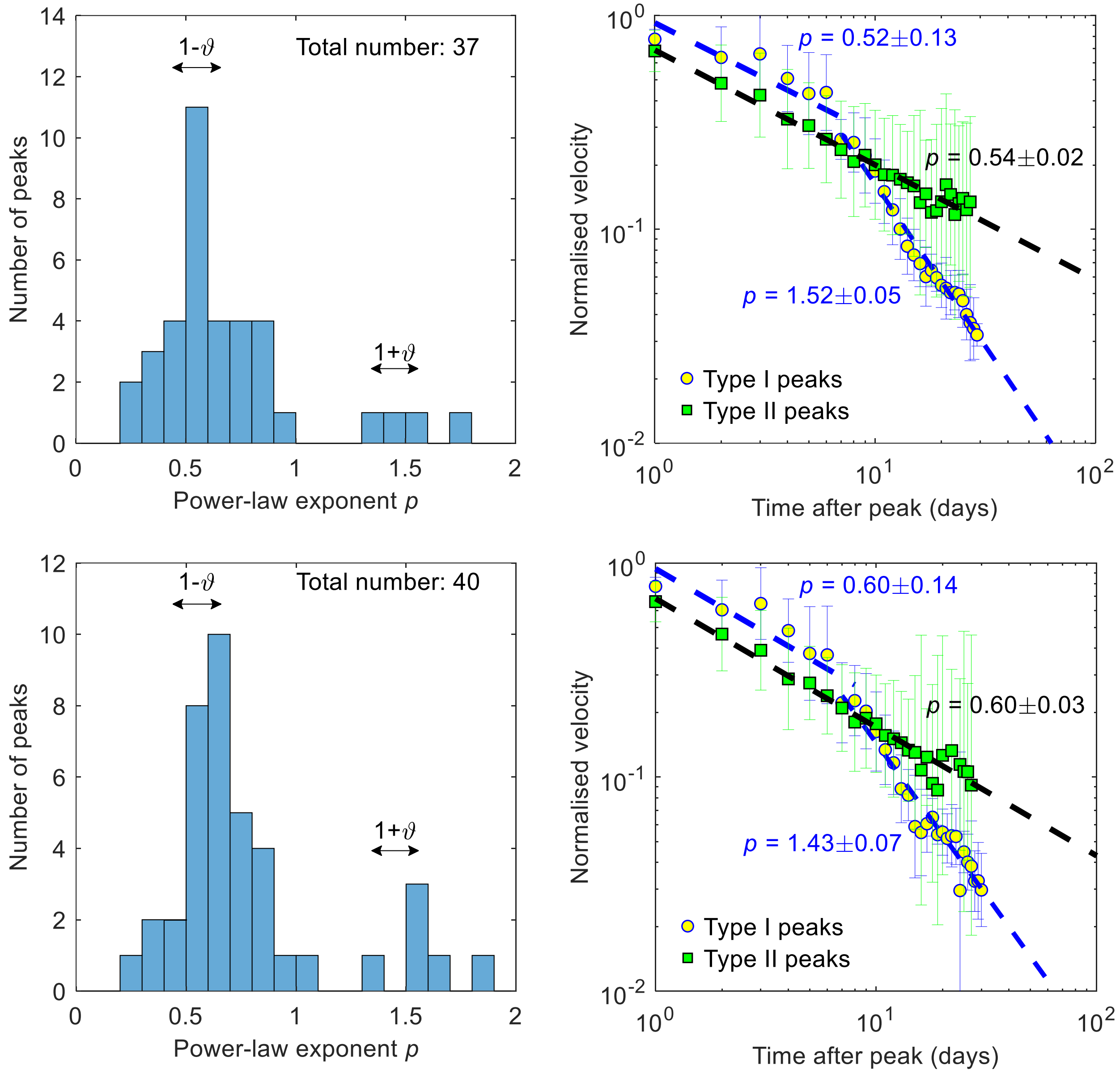

Fig. S24.

Post-peak relaxation properties associated with detected peaks in the velocity time series. Left: histogram of the power law exponents p for post-peak velocity relaxation. Right: ensemble averaged relaxation of Type I (exogenous-subcritical) and Type II (exogenous-critical) peaks. Here, a peak is qualified as a local maximum over a 40-day window which is at least $k = 2.5$ times larger than the average velocity over a 4-month (upper panel) or 10-day time window which is at least $k = 2.5$ times larger than the average velocity over a 1-month time window (lower panel), while the coefficient of determination for the fitting should meet $R^2 > 0.8$.

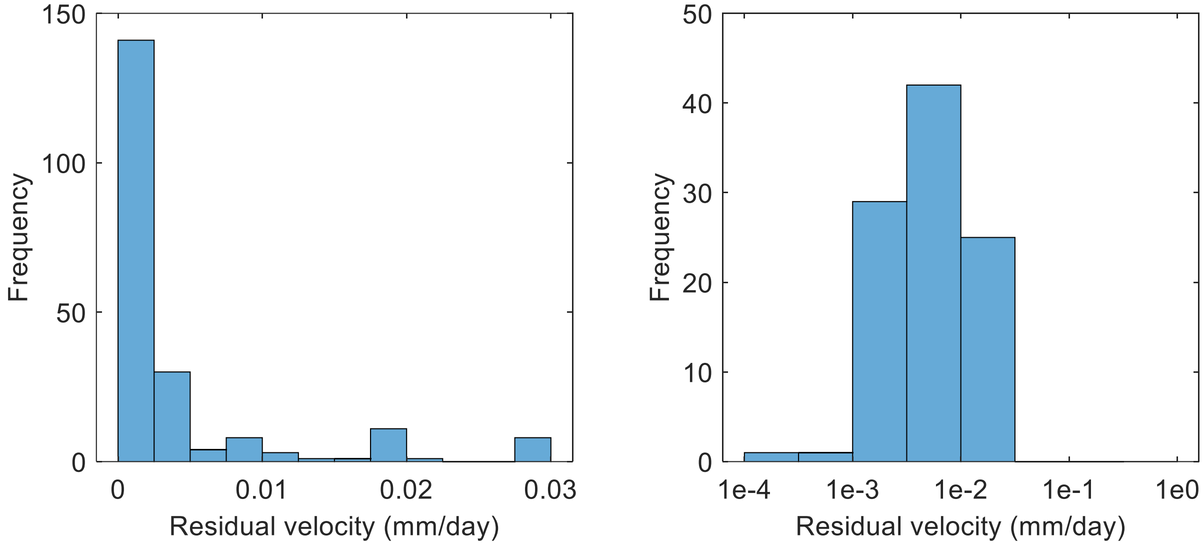

Fig. S25.

Histogram of slope residual velocities. Data are plotted in a linear scale (left) and a logarithmic scale (right).

Table S1.
Landslide information (25 landslides in total).

Site	Location	Type	Material	Failure time	Volume (m^3)	Monitoring method
Brienz (*72*)	Switzerland	Rockslide	Flysch, schists, dolomite	2023-06-15	1.2×10^6	Total station with reflectors
Grabengufer (*73*)	Switzerland	Rockfall	Rock & ice	2020-05-17	5×10^2	GNSS & inclinometer
Hogarth (*74*)	Canada	Topple	Diorite	1975-06-23	2×10^5	Extensometers
Hollin Hill (*64*)	UK	Rockslide	Sandstone, limestone, mudstone & siltstone	N/A	$\sim5\times10^3$	Shape acceleration arrays
Ingelsberg (*67*)	Austria	Rockfall	Schists	2013-04-29	20-40	Ground-based InSAR
La Saxe (*60*)	Italy	Rockslide	Meta-sedimentary sequences	2013-04-21	5×10^2-1×10^3	Total station with reflectors
La Clapière (*15*)	France	Rockslide	Metamorphic rocks	N/A	5×10^7	Distance meters
Maca (*10*)	Peru	Soilslide	Fine-grained lacustrine sediments	N/A	6×10^7	Continuous GPS stations
Moosfluh (*65*)	Switzerland	Rockslide	Metamorphic rocks	N/A	7.5×10^7	Total station with reflectors
Nevis Bluff (*75*)	New Zealand	Flexural topple / rockslide	Schist	1975-06-14	3.2×10^4	Survey markers
Oak Ridge (*7*)	USA	Earthflow	Fragments of sandstone, chert, greenstone, and blueschist	N/A	$\sim2\times10^6$	Extensometers
Pomarico (*61*)	Italy	Soilslide / earthflow	Cays & sands	N/A	4×10^6	Ground-based InSAR
Preonzo (*36*)	Switzerland	Rockslide	Gneiss	2012-05-15	2.1×10^5	Extensometers & total station with reflectors
Roesgrenda (*76*)	Norway	Soilslide	Quick clay	2000-03-02	2×10^3	Extensometers
Séchilienne (*68*)	France	Rockslide	Micaschists	N/A	$\sim4\times10^6$	Extensometers
Shuping (*66*)	PR China	Soilslide	Colluvial rubble soils & residual clayey soils	N/A	1.6×10^7	Continuous GPS stations
Tapgaon (*62*)	Nepal	Rockslide	Regolith & weathered rocks	N/A	$\sim1\times10^8$	Pléiades optical satellite images and SAR Sentinel-1 images

Table S1 (continued).

Landslide information (25 landslides in total).

Site	Location	Type	Material	Failure time	Volume (m^3)	Monitoring method
Twain Harte (*59*)	USA	Exfoliation dome (related to rockfalls)	Granite	N/A	2.3×10^3	Extensometers
Vajont (*77*)	Italy	Rockslide	Limestone	1963-10-09	2.7×10^8	Geodetic bench marks
Vallcebre (*14*)	Spain	Rockslide	Shale, gypsum & claystone	N/A	$\sim2\times10^7$	Borehole wire extensometers
Veslemannen (*58*)	Norway	Rockslide	Gneiss	2019-09-05	5.4×10^4	Ground-based InSAR
Welland (*78*)	Canada	Soilslide	Clay	1967-02-22	5×10^2	Extensometers
Xintan (*79*)	PR China	Rockslide	Sediments	1985-06-12	3×10^7	Geodetic bench marks
Yaoshan (*63*)	PR China	Soilslide	Colluvium and residual clay	N/A	5×10^5	Inclinometers
Yusuihsi (*80*)	Taiwan	Rockslide (evolved into debris flow after failure)	Slate & phyllite	2021-08-07	8.5×10^6	Optical satellite imagery

REFERENCES AND NOTES

1. D. Petley, Global patterns of loss of life from landslides. *Geology* **40**, 927–930 (2012).

2. J. Palmer, Creeping earth could hold secret to deadly landslides. *Nature* **548**, 384–386 (2017).

3. S. L. Gariano, F. Guzzetti, Landslides in a changing climate. *Earth Sci. Rev.* **162**, 227–252 (2016).

4. F. Agliardi, M. M. Scuderi, N. Fusi, C. Collettini, Slow-to-fast transition of giant creeping rockslides modulated by undrained loading in basal shear zones. *Nat. Commun.* **11**, 1352 (2020).

5. N. Bontemps, P. Lacroix, E. Larose, J. Jara, E. Taipe, Rain and small earthquakes maintain a slow-moving landslide in a persistent critical state. *Nat. Commun.* **11**, 780 (2020).

6. G. B. Crosta, C. Di Prisco, P. Frattini, G. Frigerio, R. Castellanza, F. Agliardi, Chasing a complete understanding of the triggering mechanisms of a large rapidly evolving rockslide. *Landslides* **11**, 747–764 (2014).

7. N. J. Finnegan, E. E. Brodsky, H. M. Savage, A. L. Nereson, C. R. Murphy, Seasonal slow landslide displacement is accommodated by mm-scale stick-slip events. *Geophys. Res. Lett.* **49**, e2022GL099548 (2022).

8. N. J. Finnegan, J. P. Perkins, A. L. Nereson, A. L. Handwerger, Unsaturated flow processes and the onset of seasonal deformation in slow-moving landslides. *Case Rep. Med.* **126**, e2020JF005758 (2021).

9. A. L. Handwerger, J. J. Roering, D. A. Schmidt, Controls on the seasonal deformation of slow-moving landslides. *Earth Planet. Sci. Lett.* **377-378**, 239–247 (2013).

10. P. Lacroix, H. Perfettini, E. Taipe, B. Guillier, Coseismic and postseismic motion of a landslide: Observations, modeling, and analogy with tectonic faults. *Geophys. Res. Lett.* **41**, 6676–6680 (2014).

11. N. J. Finnegan, D. M. Saffer, Seasonal slow slip in landslides as a window into the frictional rheology of creeping shear zones. *Sci. Adv.* **10**, eadq9399 (2024).

12. S. R. LaHusen, A. R. Duvall, A. M. Booth, A. Grant, B. A. Mishkin, D. R. Montgomery, W. Struble, J. J. Roering, J. Wartman, Rainfall triggers more deep-seated landslides than Cascadia earthquakes in the Oregon Coast Range, USA. *Sci. Adv.* **6**, eaba6790 (2020).

13. P. Lacroix, A. L. Handwerger, G. Bièvre, Life and death of slow-moving landslides. *Nat. Rev. Earth Environ.* **1**, 404–419 (2020).

14. J. Corominas, J. Moya, A. Lloret, J. A. Gili, M. G. Angeli, A. Pasuto, S. Silvano, Measurement of landslide displacements using a wire extensometer. *Eng. Geol.* **55**, 149–166 (2000).

15. A. Helmstetter, D. Sornette, J.-R. Grasso, J. V. Andersen, S. Gluzman, V. Pisarenko, Slider block friction model for landslides: Application to Vaiont and La Clapière landslides. *J. Geophys. Res.* **109**, 2002JB002160 (2004).

16. R. Burridge, L. Knopoff, Model and theoretical seismicity. *Bull. Seismol. Soc. Am.* **57**, 341–371 (1967).

17. J. Faillettaz, D. Sornette, M. Funk, Gravity-driven instabilities: Interplay between state- and velocity-dependent frictional sliding and stress corrosion damage cracking. *J. Geophys. Res.* **115**, doi.org/10.1029/2009JB006512 (2010).

18. D. Sornette, A. Helmstetter, Endogenous versus exogenous shocks in systems with memory. *Phys. A: Stat. Mech. Appl.* **318**, 577–591 (2003).

19. D. Sornette, "Endogenous versus exogenous origins of crises" in *Extreme Events in Nature and Society*, S. Albeverio, V. Jentsch, H. Kantz, Eds. (Springer, Berlin, Heidelberg, 2006; https://doi.org/10.1007/3-540-28611-X_5)10.1007/3-540-28611-X_5), pp. 95–119.

20. Y. Y. Kagan, L. Knopoff, Stochastic synthesis of earthquake catalogs. *J. Geophys. Res.* **86**, 2853–2862 (1981).

21. H. Perfettini, J.-P. Avouac, Postseismic relaxation driven by brittle creep: A possible mechanism to reconcile geodetic measurements and the decay rate of aftershocks, application to the Chi-Chi earthquake, Taiwan. *J. Geophys. Res.* **109**, 2003JB002488 (2004).

22. A. M. Freed, J. Lin, Delayed triggering of the 1999 Hector Mine earthquake by viscoelastic stress transfer. *Nature* **411**, 180–183 (2001).

23. M. Lindman, B. Lund, R. Roberts, K. Jonsdottir, Physics of the Omori law: Inferences from interevent time distributions and pore pressure diffusion modeling. *Tectonophys.* **424**, 209–222 (2006).

24. J. Dieterich, A constitutive law for rate of earthquake production and its application to earthquake clustering. *J. Geophys. Res.* **99**, 2601–2618 (1994).

25. A. Helmstetter, D. Sornette, Subcritical and supercritical regimes in epidemic models of earthquake aftershocks. *J. Geophys. Res.* **107**, ESE 10-1–ESE 10-21 (2002).

26. H. Nechad, A. Helmstetter, R. El Guerjouma, D. Sornette, Andrade and critical time-to-failure laws in fiber-matrix composites: Experiments and model. *J. Mech. Phys. Solids* **53**, 1099–1127 (2005).

27. C. Kisslinger, L. M. Jones, Properties of aftershock sequences in southern California. *J. Geophys. Res.* **96**, 11947–11958 (1991).

28. T. Hirata, Omori's Power Law aftershock sequences of microfracturing in rock fracture experiment. *J. Geophys. Res.* **92**, 6215–6221 (1987).

29. T. Utsu, Y. Ogata, R. S. Matsu'ura, The centenary of the Omori formula for a decay law of aftershock activity. *J. Phys. Earth* **43**, 1–33 (1995).

30. E. N. D. C. Andrade, On the viscous flow in metals, and allied phenomena. *Proc. R. Soc. Lond. A* **84**, 1–12 (1910).

31. B. E. Shaw, Generalized Omori law for aftershocks and foreshocks from a simple dynamics. *Geophys. Res. Lett.* **20**, 907–910 (1993).

32. T. E. Harris, *The Theory of Branching Processes* (Springer, Berlin, 1963).

33. D. Sornette, *Critical Phenomena in Natural Sciences - Chaos, Fractals, Selforganization and Disorder: Concepts and Tools* (Springer, Berlin/Heidelberg, 2006).

34. D. Sornette, A. Helmstetter, Occurrence of finite-time singularities in epidemic models of rupture, earthquakes, and starquakes. *Phys. Rev. Lett.* **89**, 158501 (2002).

35. J. Kemeny, The time-dependent reduction of sliding cohesion due to rock bridges along discontinuities: A fracture mechanics approach. *Rock Mech. Rock Eng.* **36**, 27–38 (2003).

36. S. Loew, S. Gschwind, V. Gischig, A. Keller-Signer, G. Valenti, Monitoring and early warning of the 2012 Preonzo catastrophic rockslope failure. *Landslides* **14**, 141–154 (2017).

37. S. Gschwind, S. Loew, A. Wolter, Multi-stage structural and kinematic analysis of a retrogressive rock slope instability complex (Preonzo, Switzerland). *Eng. Geol.* **252**, 27–42 (2019).

38. Y. Y. Kagan, Likelihood analysis of earthquake catalogues. *Geophys. J. Int.* **106**, 135–148 (1991).

39. Y. Y. Kagan, L. Knopoff, Random stress and earthquake statistics: Time dependence. *Geophys. J. Int.* **88**, 723–731 (1987).

40. S. Redner, *A Guide to First-Passage Processes* (Cambridge University Press, ed. 1, 2001; https://www.cambridge.org/core/product/identifier/9780511606014/type/book).

41. S. Nandan, S. K. Ram, G. Ouillon, D. Sornette, Is seismicity operating at a critical point? *Phys. Rev. Lett.* **126**, 128501 (2021).

42. P. Bak, *How Nature Works: The Science of Self-Organized Criticality* (Springer, New York, 1996; http://link.springer.com/10.1007/978-1-4757-5426-1).

43. D. Sornette, Predictability of catastrophic events: Material rupture, earthquakes, turbulence, financial crashes, and human birth. *Proc. Natl. Acad. Sci. U.S.A.* **99**, 2522–2529 (2002).

44. Q. Lei, D. Sornette, A stochastic dynamical model of slope creep and failure. *Geophys. Res. Lett.* **50**, e2022GL102587 (2023).

45. P. Blanc, J. Donier, J.-P. Bouchaud, Quadratic Hawkes processes for financial prices. *Quant. Finance* **17**, 171–188 (2017).

46. K. Kanazawa, D. Sornette, Asymptotic solutions to nonlinear Hawkes processes: A systematic classification of the steady-state solutions. *Phys. Rev. Res.* **5**, 013067 (2023).

47. Q. Lei, D. Sornette, H. Yang, S. Loew, Real-time forecast of catastrophic landslides via dragon-king detection. *Geophys. Res. Lett.* **50**, e2022GL100832 (2023).

48. Q. Lei, D. Sornette, Unified failure model for landslides, rockbursts, glaciers, and volcanoes. *Commun. Earth Environ.* **6**, 390 (2025).

49. Q. Lei, D. Sornette, Log-periodic power law singularities in landslide dynamics: Statistical evidence from 52 crises. *Geophys. Res. Lett.* **52**, e2025GL116379 (2025).

50. B. Voight, A method for prediction of volcanic eruptions. *Nature* **332**, 125–130 (1988).

51. E. Intrieri, T. Carlà, G. Gigli, Forecasting the time of failure of landslides at slope-scale: A literature review. *Earth Sci. Rev.* **193**, 333–349 (2019).

52. C. H. Scholz, *The Mechanics of Earthquakes and Faulting* (Cambridge University Press, ed. 3, 2019; https://www.cambridge.org/core/product/identifier/9781316681473/type/book).

53. C. H. Scholz, Mechanisms of seismic quiescences. *Pure Appl. Geophys.* **126**, 701–718 (1988).

54. R. L. Kranz, C. H. Scholz, Critical dilatant volume of rocks at the onset of Tertiary creep. *J. Geophys. Res.* **82**, 4893–4898 (1977).

55. C. H. Scholz, On the stress dependence of the earthquake *b* value. *Geophys. Res. Lett.* **42**, 1399–1402 (2015).

56. R. Ito, Y. Kaneko, Physical mechanism for a temporal decrease of the Gutenberg-Richter b-value prior to a large earthquake. *J. Geophys. Res. Solid Earth* **128**, e2023JB027413 (2023).

57. A. Helmstetter, D. Sornette, Predictability in the epidemic-type aftershock sequence model of interacting triggered seismicity. *J. Geophys. Res.* **108**, 2003JB002485 (2003).

58. L. Kristensen, J. Czekirda, I. Penna, B. Etzelmüller, P. Nicolet, J. S. Pullarello, L. H. Blikra, I. Skrede, S. Oldani, A. Abellan, Movements, failure and climatic control of the Veslemannen rockslide, Western Norway. *Landslides* **18**, 1963–1980 (2021).

59. B. D. Collins, G. M. Stock, M.-C. Eppes, S. W. Lewis, S. C. Corbett, J. B. Smith, Thermal influences on spontaneous rock dome exfoliation. *Nat. Commun.* **9**, 762 (2018).

60. A. Manconi, D. Giordan, Landslide failure forecast in near-real-time. *Geomat. Nat. Haz. Risk* **7**, 639–648 (2016).

61. L. Nava, A. Tordesillas, G. Qian, F. Catani, Displacement residuals reveal landslide regime shifts. *Landslides* **22**, 1–16 (2025).

62. P. Lacroix, T. Gavillon, C. Bouchant, J. Lavé, J.-L. Mugnier, S. Dhungel, F. Vernier, SAR and optical images correlation illuminates post-seismic landslide motion after the Mw 7.8 Gorkha earthquake (Nepal). *Sci. Rep.* **12**, 6266 (2022).

63. Z. Huang, W. Jian, Q. Liu, H. Dou, Response of step-like landslide to pore-water pressure under the action of typhoon and rainstorm. *Geofluids* **2022**, 1–13 (2022).

64. S. Uhlemann, A. Smith, J. Chambers, N. Dixon, T. Dijkstra, E. Haslam, P. Meldrum, A. Merritt, D. Gunn, J. Mackay, Assessment of ground-based monitoring techniques applied to landslide investigations. *Geomorphology* **253**, 438–451 (2016).

65. F. Glueer, S. Loew, A. Manconi, J. Aaron, From toppling to sliding: Progressive evolution of the Moosfluh landslide, Switzerland. *J. Geophys. Res. Earth Surf.* **124**, 2899–2919 (2019).

66. H. Huang, W. Yi, S. Lu, Q. Yi, G. Zhang, Use of monitoring data to interpret active landslide movements and hydrological triggers in Three Gorges Reservoir. *J. Perform. Constr. Facil.* **30**, C4014005 (2016).

67. L. Di Matteo, S. Romeo, D. S. Kieffer, Rock fall analysis in an Alpine area by using a reliable integrated monitoring system: Results from the Ingelsberg slope (Salzburg Land, Austria). *Bull. Eng. Geol. Environ.* **76**, 413–420 (2017).

68. D. Amorese, J.-R. Grasso, S. Garambois, M. Font, Change-point analysis of geophysical time-series: Application to landslide displacement rate (Séchilienne rock avalanche, France). *Geophys. J. Int.* **213**, 1231–1243 (2018).

69. C. R. J. Kilburn, D. N. Petley, Forecasting giant, catastrophic slope collapse: lessons from Vajont, Northern Italy. *Geomorphology* **54**, 21–32 (2003).

70. A. Helmstetter, Is earthquake triggering driven by small earthquakes? *Phys. Rev. Lett.* **91**, 058501 (2003).

71. V. Filimonov, S. Wheatley, D. Sornette, Effective measure of endogeneity for the autoregressive conditional duration point processes via mapping to the self-excited Hawkes process. *Commun. Nonlinear Sci. Numer. Simul.* **22**, 23–37 (2015).

72. S. Loew, S. Schneider, M. Josuran, D. Figi, R. Thoeny, A. Huwiler, A. Largiadèr, C. Naenni, Early warning and dynamics of compound rockslides: lessons learnt from the Brienz/Brinzauls rockslope failure. *Landslides* **22**, 283–298 (2025).

73. A. Cicoira, S. Weber, A. Biri, B. Buchli, R. Delaloye, R. Da Forno, I. Gärtner-Roer, S. Gruber, T. Gsell, A. Hasler, R. Lim, P. Limpach, R. Mayoraz, M. Meyer, J. Noetzli, M. Phillips, E. Pointner, H. Raetzo, C. Scapozza, T. Strozzi, L. Thiele, A. Vieli, D. Vonder Mühll, V. Wirz, J. Beutel, In situ observations of the Swiss periglacial environment using GNSS instruments. *Earth Syst. Sci. Data* **14**, 5061–5091 (2022).

74. C. O. Brawner, P. F. Stacey, "Hogarth Pit Slope Failure, Ontario, Canada" in *Developments in Geotechnical Engineering*, B. Voight, Ed. (Elsevier, 1979) vol. 14 of *Rockslides and Avalanches*, pp. 691–707.

75. I. Brown, M. Hittinger, R. Goodman, Finite element study of the Nevis Bluff (New Zealand) rock slope failure. *Rock Mech.* **12**, 231–245 (1980).

76. T. Okamoto, J. O. Larsen, S. Matsuura, S. Asano, Y. Takeuchi, L. Grande, Displacement properties of landslide masses at the initiation of failure in quick clay deposits and the effects of meteorological and hydrological factors. *Eng. Geol.* **72**, 233–251 (2004).

77. E. Nonveiller, The Vajont reservoir slope failure. *Eng. Geol.* **24**, 493–512 (1987).

78. D. Kwan, Observations of the failure of a vertical cut in clay at Welland, Ontario. *Can. Geotech. J.* **8**, 283–298 (1971).

79. L. Xue, S. Qin, P. Li, G. Li, I. Adewuyi Oyediran, X. Pan, New quantitative displacement criteria for slope deformation process: From the onset of the accelerating creep to brittle rupture and final failure. *Eng. Geol.* **182**, 79–87 (2014).

80. H.-L. Kuo, G.-W. Lin, T.-Y. Lin, C.-H. Liu, C.-R. Chu, C.-H. Chang, C.-W. Lin, H. Chen, Displacement evolution of failure and non-failure sliding rock slopes. *Landslides* **22**, 1213–1226 (2025).
